# Global Trends and Research Collaborations on Food and Beverages Warning Labels: A Bibliometric Analysis

**DOI:** 10.3390/nu16203493

**Published:** 2024-10-15

**Authors:** Akram Hernández-Vásquez, Fabriccio J. Visconti-Lopez, Rodrigo Vargas-Fernández

**Affiliations:** 1 Centro de Excelencia en Investigaciones Económicas y Sociales en Salud, Vicerrectorado de Investigación, Universidad San Ignacio de Loyola, Lima 15024, Peru; 2 Sociedad Científica de Estudiantes de Medicina—UPC, Lima 15067, Peru; fabricciovisco@gmail.com; 3 Epidemiology and Health Economics Research (EHER), Universidad Científica del Sur, Lima 15067, Peru; jvargasf@cientifica.edu.pe

**Keywords:** noncommunicable diseases, food labeling, nutrition policy, public health, bibliometrics

## Abstract

Background: Non-communicable diseases (NCDs) represent a global health challenge, accounting for 74% of deaths worldwide. One of the recommended interventions to reduce the risk of NCDs is the implementation of warning labels (WLs) on food products to alert consumers about high levels of undesirable nutrients, such as sugar or saturated fats. We aimed to conduct a bibliometric analysis of scientific literature related to WL policies in food and beverages to evaluate global trends and collaborations. Methods: A bibliometric analysis was performed using the Web of Science Core Collection (WoSCC). Articles published between 1998 and 2023 were retrieved using the search terms “warning labels” AND “food” OR “beverage”. Bibliometric indicators, including publication counts, citations, collaborations, and thematic trends, were analyzed using the Bibliometrix package in R and VOSviewer. Results: We included 255 articles on WLs. Scientific production increased markedly from 2018 onwards, with over 30 articles published annually from 2018 to 2023. The most cited article, by Taillie et al., focused on the impact of Chile’s WL policies. The United States had the largest scientific production, followed by Brazil and Chile. *Nutrients* was the journal with the most publications on this topic. Conclusions: The growth in WL-related research, particularly in Latin America, reflects the increasing implementation of these policies. These results underscore key collaborations and evolving research themes, from food labeling to broader public health impacts, emphasizing the need for continued evaluation of WL effectiveness.

## 1. Introduction

Non-communicable diseases (NCDs) are a group of conditions that accounted for approximately 41 million deaths worldwide (equivalent to 74% of all global deaths) in 2019 [1]. One of the feasible interventions found to reduce the risk of NCDs is maintaining a healthy diet [2]. Thereby, the World Health Organization (WHO) has suggested certain public health policies since 2004 to decrease the prevalence of conditions, among which is the implementation of warning labels (WLs) [3,4]. The primary goal of this strategy is to guide and raise awareness among consumers towards making healthier food choices, ultimately improving the overall quality of their diets and leading to better health outcomes [5].

A WL is a product label designed to capture attention and alert consumers when a product contains a relatively high level of an undesirable substance (e.g., sugar, salt, calories, and saturated fats, among others). This is represented by a single value and conveyed in a simple format, such as stars, colors, or a checkmark [6,7]. This public policy has been implemented in various forms across different continents, including Europe (e.g., Nordic Keyhole, Choice Program, Heart Symbol, Nutri-Score), Latin America (Warning Signs), Asia (e.g., Traffic Lights, Healthier Choice logo, Weqaya logo), Oceania (Health Star rating), and Africa (Heart Tick and Good Food logo) [5]. Evidence suggests that these types of interventions have a positive effect on consumers. A meta-analysis that included over 8600 people, both adults and children, found that WLs reduced product selection compared to the absence of graphic warning labels, resulting in participants being 26% less likely to choose a product displaying a graphic WL [8]. However, research focused on changes in the use of these policies and their implementation needs to be explored and quantified to provide a current overview of scientific production on this topic.

Understanding research trends on WLs can stimulate interest in conducting future studies on this public health policy worldwide. Therefore, a bibliometric analysis is useful as it incorporates a quantitative analysis of published articles, encompassing indicators such as the number of publications, authors, topics, and productivity over time [9]. This type of analysis is not subject to researcher biases that may occur in narrative studies, as it provides analytical, statistical, and quantitative results [10]. Conducting a bibliometric study on WLs allows for the demonstration, through statistical indicators, of whether governmental bodies and agencies are generating new evidence, especially in the context of the WHO’s recommendation of this measure [5]. We aimed to identify the bibliometric characteristics of scientific documents related to WLs. This included analyzing the number of articles, emerging trends, the most prolific authors and institutions, prominent collaborations, highly cited papers, leading journals, and the countries most active in collaborative publications, among other key factors.

## 2. Materials and Methods

### 2.1. Study Design and Information Source

A bibliometric study of scientific production on warning labels (WLs) in food and/or beverages was conducted using the Web of Science Core Collection (WoSCC). WoSCC was selected for its extensive coverage and standardization of records in multidisciplinary research.

### 2.2. Literature Collection Strategy

The literature search was executed in WoSCC on 20 February 2024, using the following terms: TS = ((warning*) AND (label*) AND (food* OR beverage* OR nutrient*)). The search strategy was developed and agreed upon by all authors.

### 2.3. Data Collection Procedure

The selection of articles was performed by one author (FJVL) after reviewing the records identified in Rayyan [11]. A second author then validated the preliminary selection. Using the selected records and their identifiers, the metadata of the included records were retrieved from WoSCC and exported to a PlainText format file. Finally, this file was imported into NotePad++ version 8.6.8 to standardize the fields for authors, affiliations, and KeyWords Plus.

### 2.4. Statistical Analysis and Data Visualization

Bibliometric indicators were analyzed using the Bibliometrix package in R (https://www.bibliometrix.org/home/ (accessed on 10 October 2024)). Moreover, VOSviewer 1.6.20 (Leiden University, Leiden, The Netherlands) [12], was employed to create networks of authors, affiliations, and keywords based on KeyWords Plus. The analysis presented the total number of articles, journals, citations, mentions, the annual number of publications, the most cited articles, the journals with the highest number of publications, and co-authorship networks based on authors, affiliations, and keywords.

Network analyses were conducted using the “fractional counting” method, which assigns fractional weights to co-authorship or keyword occurrences based on the number of articles each entity appears in. Specific limits and adjustments, such as setting a minimum threshold for author collaborations and keyword occurrences, were applied according to the recommendations in the VOSviewer version 1.6.20 manual [13].

Thematic analysis was conducted by grouping keywords that frequently co-occurred within the bibliometric network into broader categories. These categories were visually mapped into a thematic network that represents major research clusters and emerging trends in the field. The thematic map was developed by visualizing clusters and their interrelationships using VOSviewer’s clustering algorithm, which groups closely related terms into colored clusters [12].

## 3. Results

**Figure 1:**
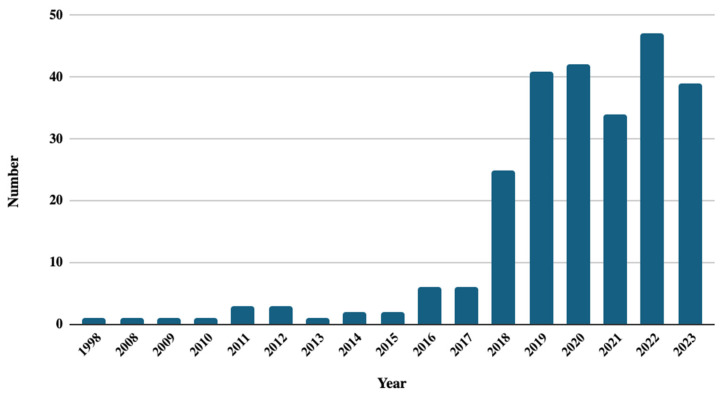
Evolution of the publication of articles on food and beverages warning labels.

A total of 255 articles on WLs were published between 1998 and 2023. While there is no defined trend in scientific production over time, a notable change is observed in 2018, where scientific production increased by 19 articles between 2017 and 2018. From 2018 onwards, scientific production exceeded 30 articles in each year up to 2023 (2019: 41, 2020: 42, 2021: 34, 2022: 47, 2023: 39) (Figure 1).

The most cited article during the study period was written by Taillie et al. [14], titled “An evaluation of Chile’s Law of Food Labeling and Advertising on sugar-sweetened beverage purchases from 2015 to 2017: A before-and-after study” and published in *PLOS Medicine* in 2020. This article has a total of 225 citations, 45 citations per year, and 8.28 normalized total citations. The second and third most cited articles were written by Corvalán et al. and published in the journal *Obesity Reviews* in 2013 (“Structural responses to the obesity and non-communicable diseases epidemic: the Chilean Law of Food Labeling and Advertising”) [15] and 2019 (“Structural responses to the obesity and non-communicable diseases epidemic: Update on the Chilean law of food labelling and advertising”) [16]. The former has a total of 160 citations, 13.33 citations per year, and 1 normalized total citation, while the latter had a lower number of citations but higher parameters of citations per year and normalized total citations (Table 1).

*Nutrients* is the journal with the highest number of publications on WLs (*n* = 39), with an impact factor of 5.9. It is considered a Q1 journal. *Public Health Nutrition* and *Frontiers in Nutrition* are the second (*n* = 16) and third (*n* = 15) journals with the highest number of publications on WLs, respectively. *Public Health Nutrition* has an impact factor of 3.2 and is a Q3 journal, while *Frontiers in Nutrition* has an impact factor of 5 and is situated in Q2. The journals ranked between fourth and tenth place are from the United Kingdom, the United States, and Switzerland, while in subsequent positions are the *Chilean Journal of Nutrition* and the *Pan American Journal of Public Health* (Table 2).

According to the countries of the corresponding authors, the United States has the highest scientific production on WLs (*n* = 50), with 36 single-country publications (SCP) and 62 multiple-country publications (MCP). Moreover, the countries ranked between second and fifth place are from the Latin America and Caribbean region. Brazil is the country with the second highest production (*n* = 32), followed by Chile (*n* = 30), Uruguay (*n* = 25), and Mexico (*n* = 16) (Table 3).

**Figure 2:**
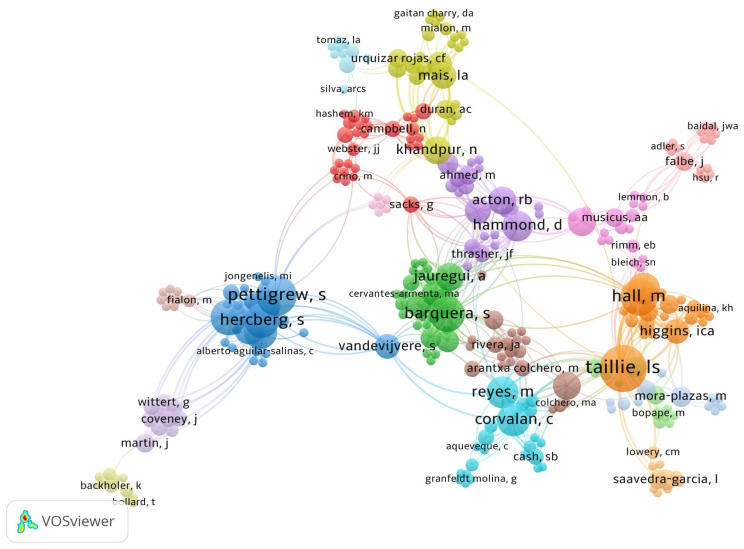
Network analysis of authors co-authorship on food and beverages warning labels.

Figure 2 shows the network of authors who have published articles on WLs, highlighting that authors with the highest scientific production were Taillie LS, Hall M, Pettigrew S, Hercberg S, Barquera S, Hammond D, Acton RB, Jáuregui A, Reyes M, and Corvalán C. Furthermore, the largest author networks are observed with authors such as Barquera S, Pettigrew S, Hercberg S, Taillie LS, Hall M, Reyes M, Corvalán C, Hammond D, Acton RB, and Vandevijvere S.

**Figure 3:**
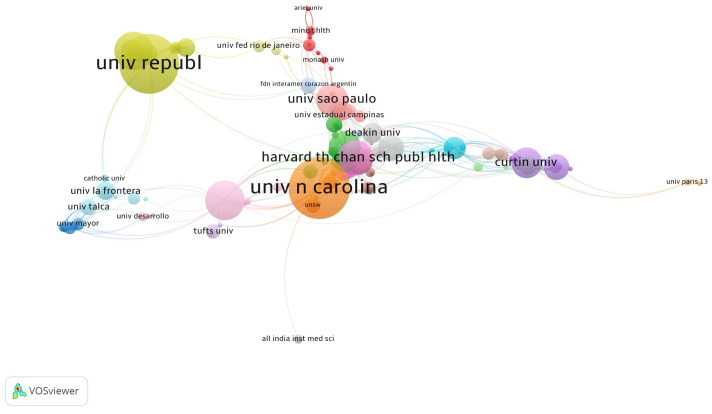
Network analysis of organization co-authorship on food and beverages warning labels.

Within the institutional network, the University of North Carolina and the Universidad La República de Chile have the highest scientific production on WLs. Moreover, universities located in Chile such as Universidad La Frontera, the Universidad de Talca, the Universidad Católica de Chile, and Universidad Mayor de Chile have collaborative networks with the Universidad La República de Chile due to territorial proximity and with the University of North Carolina. Furthermore, universities such as Harvard University in the United States, the Universidade de São Paulo in Brazil, and Curtin University in Australia have notable scientific production and collaborative networks with various universities around the world (Figure 3).

**Figure 4:**
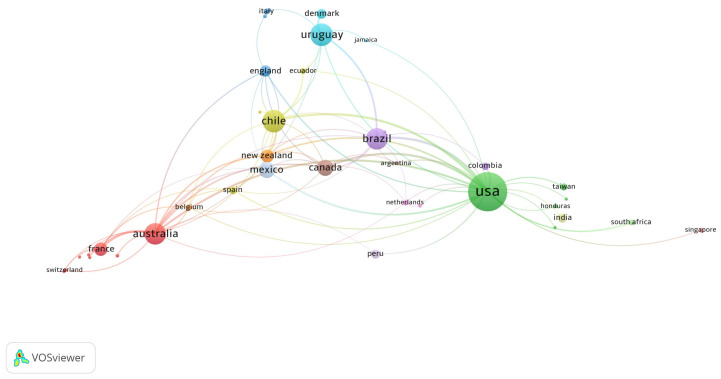
Network analysis of countries co-authorship on food and beverages warning labels.

Regarding the country network, the United States has the highest production on WLs and a collaborative network with various countries located in different regions of the world. Moreover, South American countries such as Chile, Brazil, and Uruguay have a collaborative network with various countries, including the United States. Specifically, the strongest collaborative network is observed between the United States and Chile. Furthermore, countries such as Canada, Mexico, New Zealand, and Australia have lower production but collaborative networks with the aforementioned countries (Figure 4).

**Figure 5:**
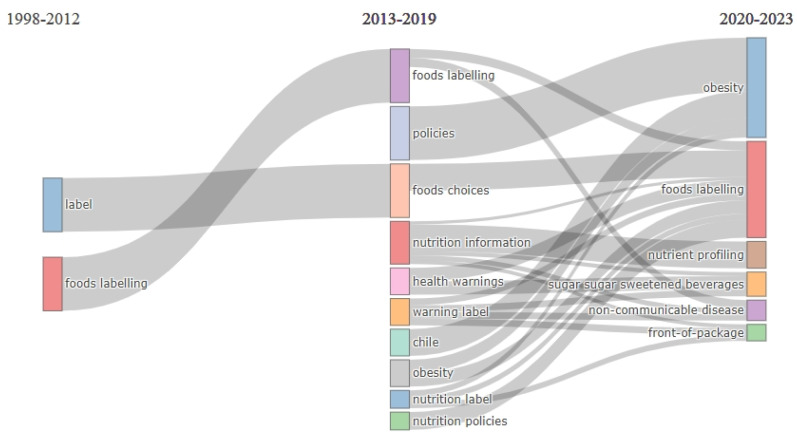
Thematic evolution of research on food and beverages warning labels.

Figure 5 shows the thematic evolution of WL research. In the period from 1998 to 2012, the most researched topics were labels and food labeling. In a subsequent period (2013–2019), an increase in studied topics was found, including policies, food choices, nutrition information, health warnings, warning labels, Chile, obesity, nutrition labels, and nutrition policies. Moreover, among the topics investigated in that period, the themes with the highest production were food labeling, policies, and food choices. In the last period, from 2020 to 2023, the most studied types were obesity and food labeling, and new topics such as nutrient profiling, sugar-sweetened beverages, non-communicable diseases, and front-of-package were added.

**Figure 6:**
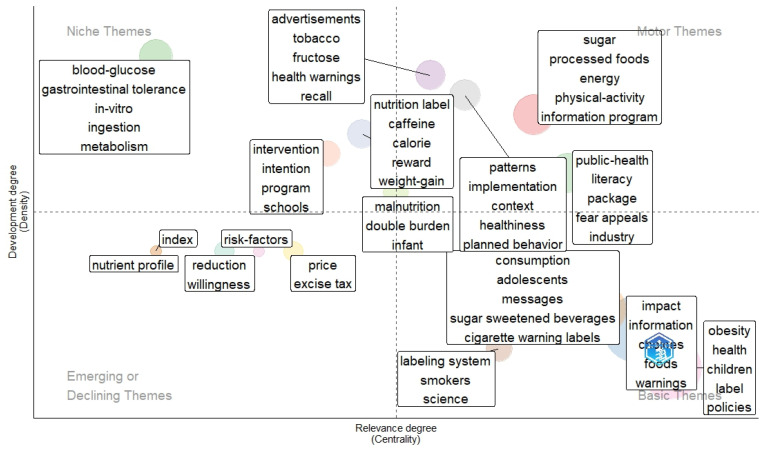
Thematic map of research on food and beverages warning labels.

Figure 6 shows the thematic map of research on WLs. The most relevant and developed themes (motor themes) are related to sugar, processed foods, energy, physical activity, information programs, public health, literacy, packaging, patterns, implementation, context, advertisements, tobacco, fructose, health warnings, and recall. The emerging or declining themes were indexes, risk factors, nutrient profiles, reduction willingness, and price excise taxes. On the other hand, the most relevant niche themes were blood glucose, gastrointestinal tolerance, in-vitro, ingestion, metabolism, intervention, intention, programs, and schools. More details can be observed in Figure 6.

## 4. Discussion

Our study aimed to evaluate the bibliometric characteristics of scientific production on WLs in the WoSCC. A total of 255 documents published up to February 2024 were identified. During the study period, the most cited article, by Taillie et al. and published in *PLOS Medicine* in 2020, accumulated 225 citations [14]. The next most cited articles were by Corvalán et al. in *Obesity Reviews* in 2013 and 2019, with 160 and 153 citations, respectively [15,16]. The United States had the highest scientific production on the topic, followed by Latin American countries such as Brazil, Chile, Uruguay, and Mexico. The collaboration network between countries showed a collaboration between the United States and Chile. The thematic evolution revealed a shift in research topics over time, from food labeling to broader themes such as food policies and non-communicable diseases. Lastly, the thematic map highlighted topics such as sugar content, processed foods, and physical activity as the most relevant in the field of warning labeling.

A change in the increase of articles on WLs was evidenced during 2018 and 2019. This change could correlate with the implementation of new policies developed before and during this period. In Chile, Ecuador, Bolivia, Uruguay, and Peru, regulations related to nutritional warnings have been promulgated [34,35,36,37], most of which are mandatory. These regulations are focused on regulating the advertising and consumption of non-alcoholic food and beverages through WLs. The evaluation of the impact of these regulations on nutritional outcomes has allowed for the elaboration and publication of various studies. The results of these studies have shown that these measures were associated with better understanding of nutritional information, generating a negative perception towards unhealthy products or positive attitudes towards healthy foods [38]. Consequently, the increase in the number of articles could be directly related to the rapid implementation of policies and their evaluation through publications, being more prominent in the United States and Latin America [39], reflecting a multinational movement towards greater awareness and regulation in promoting healthy eating.

The article written by Taillie et al. published in *PLoS Medicine* in 2020 was the most cited study and examined the impact of Chile’s 2015 and 2017 food policies on the purchase of beverages high in sugar, saturated fat, sodium, and calories, using national household-level food purchase data [14]. The study reported a 23.7% decrease in the purchase volume of these beverages after the implementation of the regulation, equivalent to 22.8 mL per capita per day. The authors concluded that after Chile implemented the Food Labeling and Advertising Law, there was a notable reduction in purchases of beverages with unhealthy ingredients. The second and third most cited articles were written by Corvalán et al. The first describes the Chilean government’s response to the obesity and non-communicable disease epidemic, focused on the Food Labeling and Advertising Laws [15]. This law sought to improve consumer information and reduce children’s exposure to unhealthy foods. The authors mention that, despite resistance from the food industry (e.g., media, politicians, and companies stating that the law violates freedom of expression, among others), the implementation of regulatory norms is still expected (e.g., cut-off points for implementing nutritional alerts and adequate size of WLs, among others) [15]. The second commented on the updating of this law, indicating new implementations of regulatory norms, such as the prohibition of the sale of regulated foods in specific places and their promotion aimed at children under 14 years of age [16]. The authors concluded that these regulatory measures are headed in the right direction but will need to be continued and complemented to achieve the desired effect of halting the obesity epidemic.

Lindsey Smith Taillie, an American epidemiologist at the University of North Carolina at Chapel Hill (the institution with the highest number of articles in this field), stands out as the most prolific author. Alongside her colleague Marissa Hall, who holds the second-highest number of publications, their research focuses on designing and evaluating food policies aimed at promoting healthier, more sustainable, and equitable diets globally [40,41,42]. Both, Taillie and Hall are members of the Global Food Research Program, a large multinational project to inform and evaluate healthy food policies worldwide, with a primary focus on Latin America [43]. Their collaboration extends to prominent Latin American researchers, such as Simon Barquera and Alejandra Jáuregui (both from the National Institute of Public Health, Ciudad de Mexico, Mexico), Marcela Reyes, and Camila Corvalán (both from the Universidad de Chile, Chile) [44,45]. On the other hand, other leading contributors to the field include Simone Pettigrew from The George Institute, Barangaroo, Australia and Serge Hercberg from Université Sorbonne Paris Nord, Paris, France, both of whom collaborate closely [46,47]. Additionally, David Hammond and Rachel Acton, who work at the University of Waterloo, Waterloo, Canada, are also key figures in this area of research [32,48].

In terms of thematic evolution, a shift from broader topics to more specific issues can be observed. The first keywords in the studies (labels and foods labelling) focused on characterizing and defining WLs as front-of-pack logos, which were considered more effective in guiding consumers toward healthy choices compared to traditional nutritional information found on the back of packaging [49,50,51]. Over time, keywords related to the development of policies (e.g., policies and nutrition policies) began to surface in various countries [39,52,53,54]. Currently, the focus has shifted towards non-communicable diseases (NCDs), which is likely due to the strong association between NCDs (e.g., heart disease, stroke, obesity, diabetes, among others) and high-calorie diets. WLs have proven to be an effective measure in reducing the consumption of unhealthy foods, thus helping to mitigate the risk and prevalence of these diseases [55,56,57]. In this sense, the World Health Organization has implemented multiple strategies aimed at reducing the incidence and burden of NCDs [58], where WLs are one of the main strategies to decrease these indicators.

There are some limitations in the present study. One of them is that this type of study does not produce a hypothesis, but only provides a description of articles on a topic; however, this type of design is useful to see if government agencies and organizations are generating new evidence and guiding future research. Moreover, the present study was based on only one database, which may leave out important sources of information; however, WoSCC is one of the largest bibliographic databases that includes the indexing of scientific publications from different thematic areas [59], including nutrition, and has ideal tools for downloading metadata for analysis [60,61,62]. Furthermore, bibliometric studies can provide data on the quantity of publications in a given area but may lack contextual information about the quality or relevance of those works. Finally, the inclusion of only English-written studies may have led to the underrepresentation of non-English research and affected the full picture of global trends.

## 5. Conclusions

The bibliometric analysis reveals a growth in scientific production on WLs from 2018, with a notable increase in the number of articles published, due to the elaboration and integration of new laws in the field. The research also indicates that the highest productivity is in Latin America, with the alliance between the United States and Chile being the largest on this topic. A progressive change in the topics addressed in the research can also be noted, reflecting an evolution in approaches over time. Initially, it focused on fundamental aspects such as food labeling, then delved into more complex areas such as obesity and non-communicable diseases, which could evidence the importance and need to continue researching and evaluating policies related to warning labels to address public health challenges associated with diet and nutrition.

## Figures and Tables

**Table 1:** Top 20 most cited articles on food and beverages warning labels.

*n*	Author and Year	Journal	TC	TC per Year	NTC
1	Taillie LS, 2020 [14]	*PLoS Med.*	225	45	8.28
2	Corvalán C, 2013 [15]	*Obes. Rev.*	160	13.33	1
3	Corvalán C, 2019 [16]	*Obes. Rev.*	153	25.5	4.38
4	Egnell M, 2018 [17]	*Nutrients*	148	21.14	3.09
5	Khandpur N, 2018 [18]	*Nutrients*	119	17	2.49
6	Arrúa A, 2017 [19]	*Appetite*	119	14.88	1.8
7	Arrúa A, 2017 [20]	*Public Health Nutr.*	116	14.5	1.76
8	Roberto CA, 2016 [21]	*Pediatrics*	114	12.67	1.74
9	Correa T, 2019 [22]	*Int. J. Behav. Nutr. Phys.*	112	18.67	3.2
10	Reyes M, 2019 [23]	*BMC Public Health*	106	17.67	3.03
11	Reyes M, 2020 [24]	*PLoS Med.*	101	20.2	3.72
12	Bollard T, 2016 [25]	*Int. J. Behav. Nutr. Phys.*	101	11.22	1.54
13	Liem DG, 2012 [26]	*Foods Qual. Prefer.*	91	7	1.99
14	Ares G, 2018 [27]	*Foods Qual. Prefer.*	84	12	1.76
15	VanEpps EM, 2016 [28]	*Am. J. Prev. Med.*	82	9.11	1.25
16	Donnelly GE, 2018 [29]	*Psychol. Sci.*	77	11	1.61
17	Machín L, 2018 [30]	*Appetite*	77	11	1.61
18	Neal B, 2017 [31]	*Nutrients*	74	9.25	1.12
19	Acton RB, 2019 [32]	*Int. J. Behav. Nutr. Phys.*	73	12.17	2.09
20	Taillie LS, 2021 [33]	*Lancet Planet. Health*	72	18	5.31

TC: total citations. NTC: normalized total citation.

**Table 2:** Top 20 journals with the highest number of publications on food and beverages warning labels.

*n*	Journal	Articles	Journal Impact Factor *	Journal Quartile *
1	*Nutrients*	39	5.9	Q1
2	*Public Health Nutrition*	16	3.2	Q3
3	*Frontiers* *in Nutrition*	15	5	Q2
4	*Food Quality* *and Preference*	12	5.3	Q1
5	*International Journal* *of Behavioral Nutrition* *and Physical Activity*	11	8.7	Q1
6	*PLoS ONE*	11	3.7	Q2
7	*Appetite*	9	5.4	Q2
8	*PLOS Medicine*	9	15.8	Q1
9	*B* *MC* *Public Health*	8	4.5	Q2
10	*International Journal of Environmental Research and Public Health*	7	4.6	Q2
11	*American Journal of Preventive Medicine*	6	5.5	Q1
12	*Food Research International*	6	8.1	Q1
13	*Revista Chilena* *de Nutrición*	5	0.7	NA
14	*Archives* *of Public Health*	4	3.3	Q2
15	*Frontiers* *in Public Health*	4	5.2	Q1
16	*Journal* *of Nutrition Education* *and Behavior*	4	2.6	Q3
17	*Revista Panamericana de Salud Publica*	4	2.6	Q3
18	*B* *MJ Open*	3	2.9	Q2
19	*Foods*	3	5.2	Q1
20	*Obesity Reviews*	3	8.9	Q1

* From Journal Citation Reports 2022. NA: not available.

**Table 3:** Top 20 countries of authors with publications on food and beverages warning labels.

*n*	Country	Articles	Freq	SCP	MCP	MCP Ratio
1	USA	50	0.201	36	14	0.28
2	Brazil	32	0.128	13	19	0.594
3	Chile	30	0.12	16	14	0.467
4	Uruguay	25	0.1	14	11	0.44
5	Mexico	16	0.064	11	5	0.312
6	Canada	14	0.056	9	5	0.357
7	Australia	11	0.044	4	7	0.636
8	France	10	0.04	0	10	1
9	United Kingdom	8	0.032	6	2	0.25
10	China	7	0.028	3	4	0.571
11	India	6	0.024	5	1	0.167
12	Peru	6	0.024	5	1	0.167
13	Italy	4	0.016	2	2	0.5
14	Colombia	3	0.012	1	2	0.667
15	New Zealand	3	0.012	3	0	0
16	Singapore	3	0.012	2	1	0.333
17	South Africa	3	0.012	0	3	1
18	Belgium	2	0.008	0	2	1
19	Germany	2	0.008	2	0	0
20	Honduras	2	0.008	1	1	0.5

SCP: single-country publications. MCP: multiple-country publications.

## Data Availability

The original data presented in the study were obtained from Web of Science. A list of manually selected articles is provided in Supplementary Material.

## Supplementary Materials

The following supporting information can be downloaded at: https://www.mdpi.com/article/10.3390/nu16203493/s1, File S1: List of manually selected articles.

## References

[1] World Health Organization (2022). Invisible Numbers: The True Extent of Noncommunicable Diseases and What to Do about Them.

[2] World Health Organization (2018). Saving Lives, Spending Less: A Strategic Response to Noncommunicable Diseases.

[3] Kanter R., Vanderlee L., Vandevijvere S. (2018). Front-of-Package Nutrition Labelling Policy: Global Progress and Future Directions. Public Health Nutr..

[4] Khaltaev N., Axelrod S. (2023). Countrywide “Best Buy” Interventions for Noncommunicable Diseases Prevention and Control in Countries with Different Level of Socioeconomic Development. Chronic Dis. Transl. Med..

[5] Jones A., Neal B., Reeve B., Ni Mhurchu C., Thow A.M. (2019). Front-of-Pack Nutrition Labelling to Promote Healthier Diets: Current Practice and Opportunities to Strengthen Regulation Worldwide. BMJ Glob. Health.

[6] Temple N.J. (2020). Front-of-Package Food Labels: A Narrative Review. Appetite.

[7] Braesco V., Drewnowski A. (2023). Are Front-of-Pack Nutrition Labels Influencing Food Choices and Purchases, Diet Quality, and Modeled Health Outcomes? A Narrative Review of Four Systems. Nutrients.

[8] Clarke N., Pechey E., Kosīte D., König L.M., Mantzari E., Blackwell A.K.M., Marteau T.M., Hollands G.J. (2021). Impact of Health Warning Labels on Selection and Consumption of Food and Alcohol Products: Systematic Review with Meta-Analysis. Health Psychol. Rev..

[9] Donthu N., Kumar S., Mukherjee D., Pandey N., Lim W.M. (2021). How to Conduct a Bibliometric Analysis: An Overview and Guidelines. J. Bus. Res..

[10] Moed H.F. (2009). New Developments in the Use of Citation Analysis in Research Evaluation. Arch. Immunol. Ther. Exp..

[11] Ouzzani M., Hammady H., Fedorowicz Z., Elmagarmid A. (2016). Rayyan—A Web and Mobile App for Systematic Reviews. Syst. Rev..

[12] Van Eck N.J., Waltman L. (2010). Software Survey: VOSviewer, a Computer Program for Bibliometric Mapping. Scientometrics.

[13] van Eck N.J., Waltman L. (2023). Manual for VOSviewer Version 1.6.20.

[14] Taillie L.S., Reyes M., Colchero M.A., Popkin B., Corvalán C. (2020). An Evaluation of Chile’s Law of Food Labeling and Advertising on Sugar-Sweetened Beverage Purchases from 2015 to 2017: A before-and-after Study. PLoS Med..

[15] Corvalán C., Reyes M., Garmendia M.L., Uauy R. (2013). Structural Responses to the Obesity and Non-Communicable Diseases Epidemic: The Chilean Law of Food Labeling and Advertising. Obes. Rev..

[16] Corvalán C., Reyes M., Garmendia M.L., Uauy R. (2019). Structural Responses to the Obesity and Non-communicable Diseases Epidemic: Update on the Chilean Law of Food Labelling and Advertising. Obes. Rev..

[17] Egnell M., Talati Z., Hercberg S., Pettigrew S., Julia C. (2018). Objective Understanding of Front-of-Package Nutrition Labels: An International Comparative Experimental Study across 12 Countries. Nutrients.

[18] Khandpur N., Sato P.D.M., Mais L.A., Martins A.P.B., Spinillo C.G., Garcia M.T., Rojas C.F.U., Jaime P.C. (2018). Are Front-of-Package Warning Labels More Effective at Communicating Nutrition Information than Traffic-Light Labels? A Randomized Controlled Experiment in a Brazilian Sample. Nutrients.

[19] Arrúa A., Curutchet M.R., Rey N., Barreto P., Golovchenko N., Sellanes A., Velazco G., Winokur M., Giménez A., Ares G. (2017). Impact of Front-of-Pack Nutrition Information and Label Design on Children’s Choice of Two Snack Foods: Comparison of Warnings and the Traffic-Light System. Appetite.

[20] Arrúa A., Machín L., Curutchet M.R., Martínez J., Antúnez L., Alcaire F., Giménez A., Ares G. (2017). Warnings as a Directive Front-of-Pack Nutrition Labelling Scheme: Comparison with the Guideline Daily Amount and Traffic-Light Systems. Public Health Nutr..

[21] Roberto C.A., Wong D., Musicus A., Hammond D. (2016). The Influence of Sugar-Sweetened Beverage Health Warning Labels on Parents’ Choices. Pediatrics.

[22] Correa T., Fierro C., Reyes M., Dillman Carpentier F.R., Taillie L.S., Corvalan C. (2019). Responses to the Chilean Law of Food Labeling and Advertising: Exploring Knowledge, Perceptions and Behaviors of Mothers of Young Children. Int. J. Behav. Nutr. Phys. Act..

[23] Reyes M., Garmendia M.L., Olivares S., Aqueveque C., Zacarías I., Corvalán C. (2019). Development of the Chilean Front-of-Package Food Warning Label. BMC Public Health.

[24] Reyes M., Smith Taillie L., Popkin B., Kanter R., Vandevijvere S., Corvalán C. (2020). Changes in the Amount of Nutrient of Packaged Foods and Beverages after the Initial Implementation of the Chilean Law of Food Labelling and Advertising: A Nonexperimental Prospective Study. PLoS Med..

[25] Bollard T., Maubach N., Walker N., Ni Mhurchu C. (2016). Effects of Plain Packaging, Warning Labels, and Taxes on Young People’s Predicted Sugar-Sweetened Beverage Preferences: An Experimental Study. Int. J. Behav. Nutr. Phys. Act..

[26] Liem D.G., Toraman Aydin N., Zandstra E.H. (2012). Effects of Health Labels on Expected and Actual Taste Perception of Soup. Food Qual. Prefer..

[27] Ares G., Varela F., Machin L., Antúnez L., Giménez A., Curutchet M.R., Aschemann-Witzel J. (2018). Comparative Performance of Three Interpretative Front-of-Pack Nutrition Labelling Schemes: Insights for Policy Making. Food Qual. Prefer..

[28] VanEpps E.M., Roberto C.A. (2016). The Influence of Sugar-Sweetened Beverage Warnings. Am. J. Prev. Med..

[29] Donnelly G.E., Zatz L.Y., Svirsky D., John L.K. (2018). The Effect of Graphic Warnings on Sugary-Drink Purchasing. Psychol. Sci..

[30] Machín L., Aschemann-Witzel J., Curutchet M.R., Giménez A., Ares G. (2018). Does Front-of-Pack Nutrition Information Improve Consumer Ability to Make Healthful Choices? Performance of Warnings and the Traffic Light System in a Simulated Shopping Experiment. Appetite.

[31] Neal B., Crino M., Dunford E., Gao A., Greenland R., Li N., Ngai J., Ni Mhurchu C., Pettigrew S., Sacks G. (2017). Effects of Different Types of Front-of-Pack Labelling Information on the Healthiness of Food Purchases—A Randomised Controlled Trial. Nutrients.

[32] Acton R.B., Jones A.C., Kirkpatrick S.I., Roberto C.A., Hammond D. (2019). Taxes and Front-of-Package Labels Improve the Healthiness of Beverage and Snack Purchases: A Randomized Experimental Marketplace. Int. J. Behav. Nutr. Phys. Act..

[33] Taillie L.S., Bercholz M., Popkin B., Reyes M., Colchero M.A., Corvalán C. (2021). Changes in Food Purchases after the Chilean Policies on Food Labelling, Marketing, and Sales in Schools: A before and after Study. Lancet Planet. Health.

[34] Köncke F., Toledo C., Berón C., Carriquiry A., Köncke F., Toledo C., Berón C., Carriquiry A. (2021). El consumo de productos ultraprocesados y su impacto en el perfil alimentario de los escolares uruguayos. Arch. Pediatr. Urug..

[35] Arista Fernández H., Mundaca Rojas K.G., Sosa Flores J., Torres Anaya V. (2018). Ley 30021 de Promoción de Alimentación Saludable Para Niños, Niñas y Adolescentes. Salud Colect..

[36] Trejo Osti L.E., Ramírez Moreno E., Ruvalcaba Ledezma J.C. (2021). Efecto del etiquetado frontal de advertencia de alimentos y bebidas. La experiencia de otros países de América Latina. J. Negat. Posit. Results.

[37] Rodríguez Osiac L., Pizarro Quevedo T. (2018). Law of Food Labelling and Advertising: Chile innovating in public nutrition once again. Rev. Chil. Pediatr..

[38] Song J., Brown M.K., Tan M., MacGregor G.A., Webster J., Campbell N.R.C., Trieu K., Ni Mhurchu C., Cobb L.K., He F.J. (2021). Impact of Color-Coded and Warning Nutrition Labelling Schemes: A Systematic Review and Network Meta-Analysis. PLoS Med..

[39] World Health Organization (2024). The Global Food Research Program at the University of North Carolina at Chapel Hill Front-of-Package Labels around the World.

[40] Taillie L.S., Hall M.G., Popkin B.M., Ng S.W., Murukutla N. (2020). Experimental Studies of Front-of-Package Nutrient Warning Labels on Sugar-Sweetened Beverages and Ultra-Processed Foods: A Scoping Review. Nutrients.

[41] Grummon A.H., Hall M.G., Taillie L.S., Brewer N.T. (2019). How Should Sugar-Sweetened Beverage Health Warnings Be Designed? A Randomized Experiment. Prev. Med..

[42] Hall M.G., Lazard A.J., Grummon A.H., Mendel J.R., Taillie L.S. (2020). The Impact of Front-of-Package Claims, Fruit Images, and Health Warnings on Consumers’ Perceptions of Sugar-Sweetened Fruit Drinks: Three Randomized Experiments. Prev. Med..

[43] (2024). The Global Food Research Program at the University of North Carolina at Chapel Hill About. Global Food Research Program. https://www.globalfoodresearchprogram.org/.

[44] Popkin B.M., Barquera S., Corvalan C., Hofman K.J., Monteiro C., Ng S.W., Swart E.C., Taillie L.S. (2021). Towards Unified and Impactful Policies to Reduce Ultra-Processed Food Consumption and Promote Healthier Eating. Lancet Diabetes Endocrinol..

[45] Roberto C.A., Ng S.W., Ganderats-Fuentes M., Hammond D., Barquera S., Jauregui A., Taillie L.S. (2021). The Influence of Front-of-Package Nutrition Labeling on Consumer Behavior and Product Reformulation. Annu. Rev. Nutr..

[46] Talati Z., Egnell M., Hercberg S., Julia C., Pettigrew S. (2019). Consumers’ Perceptions of Five Front-of-Package Nutrition Labels: An Experimental Study Across 12 Countries. Nutrients.

[47] Talati Z., Egnell M., Hercberg S., Julia C., Pettigrew S. (2019). Food Choice Under Five Front-of-Package Nutrition Label Conditions: An Experimental Study Across 12 Countries. Am. J. Public Health.

[48] Acton R.B., Hammond D. (2018). The Impact of Price and Nutrition Labelling on Sugary Drink Purchases: Results from an Experimental Marketplace Study. Appetite.

[49] Geiger C.J., Wyse B.W., Michael Parent C.R., Gaurth Hansen R. (1991). Review of Nutrition Labeling Formats. J. Am. Diet. Assoc..

[50] Feunekes G.I.J., Gortemaker I.A., Willems A.A., Lion R., Van Den Kommer M. (2008). Front-of-Pack Nutrition Labelling: Testing Effectiveness of Different Nutrition Labelling Formats Front-of-Pack in Four European Countries. Appetite.

[51] Van Kleef E., Van Trijp H., Paeps F., Fernández-Celemín L. (2008). Consumer Preferences for Front-of-Pack Calories Labelling. Public Health Nutr..

[52] Watson W.L., Kelly B., Hector D., Hughes C., King L., Crawford J., Sergeant J., Chapman K. (2014). Can Front-of-Pack Labelling Schemes Guide Healthier Food Choices? Australian Shoppers’ Responses to Seven Labelling Formats. Appetite.

[53] Gregori D., Ballali S., Vögele C., Gafare C.E., Stefanini G., Widhalm K. (2014). Evaluating Food Front-of-Pack Labelling: A Pan-European Survey on Consumers’ Attitudes toward Food Labelling. Int. J. Food Sci. Nutr..

[54] Devi A., Eyles H., Rayner M., Ni Mhurchu C., Swinburn B., Lonsdale-Cooper E., Vandevijvere S. (2014). Nutritional Quality, Labelling and Promotion of Breakfast Cereals on the New Zealand Market. Appetite.

[55] Sagaceta-Mejía J., Tolentino-Mayo L., Cruz-Casarrubias C., Nieto C., Barquera S. (2022). Understanding of Front of Package Nutrition Labels: Guideline Daily Amount and Warning Labels in Mexicans with Non-Communicable Diseases. PLoS ONE.

[56] Saleem S.M., Bhattacharya S., Deshpande N. (2022). Non-Communicable Diseases, Type 2 Diabetes, and Influence of Front of Package Nutrition Labels on Consumer’s Behaviour: Reformulations and Future Scope. Diabetes Metab. Syndr. Clin. Res. Rev..

[57] Pan American Health Organization (2020). Pan American Health Organization Front-of-Package Labeling as a Policy Tool for the Prevention of Noncommunicable Diseases in the Americas.

[58] WHO, World Health Organization, FAO (2003). Diet, Nutrition, and the Prevention of Chronic Diseases: Report of a WHO-FAO Expert Consultation; [Joint WHO-FAO Expert Consultation on Diet, Nutrition, and the Prevention of Chronic Diseases; WHO: Geneva, Switzerland, 2002].

[59] Birkle C., Pendlebury D.A., Schnell J., Adams J. (2020). Web of Science as a Data Source for Research on Scientific and Scholarly Activity. Quant. Sci. Stud..

[60] Kristia K., Kovács S., Bács Z., Rabbi M.F. (2023). A Bibliometric Analysis of Sustainable Food Consumption: Historical Evolution, Dominant Topics and Trends. Sustainability.

[61] Ungureanu E.L., Mocanu A.L., Stroe C.A., Duță D.E., Mustățea G. (2023). Assessing Health Risks Associated with Heavy Metals in Food: A Bibliometric Analysis. Foods.

[62] Dzhunushalieva G.D., Teuber R. (2024). A Bibliometric Analysis of Trends in the Relationship between Innovation and Food. BFJ.

