# Supplementary material for: Global Trends and Research Collaborations on Food and Beverages Warning Labels: A Bibliometric Analysis
Source: Nutrients. 2024 Oct 15;16(20):3493. doi: 10.3390/nu16203493 (PMC11510383; doi:10.3390/nu16203493)
Supplement: Supplementary file 1 [file nutrients-16-03493-s001.zip › nutrients-3226047-supplementary.pdf]

File S1: List of manually selected articles

1. Ares, G.; Aschemann-Witzel, J.; Rosa Curutchet, M.; Antunez, L.; Moratorio, X.; Bove, I. A Citizen Perspective on Nutritional Warnings as Front-of-Package Label: Insights for the Design of Accompanying Policies Measures. PUBLIC Health Nutr. 2018, 21, 3450–3461, doi:10.1017/S1368980018002045.

2. Khandpur, N.; Mais, L.A.; Bortoletto Martins, A.P. A Comparative Assessment of Two Different Front-of-Package Nutrition Label Designs: A Randomized Experiment in Brazil. PLoS ONE 2022, 17, e0265990, doi:10.1371/journal.pone.0265990.

3. Blumenberg, A. A Cross-Sectional Assessment of Package Label Aimed at Children of Edible and Leaf Cannabis Discarded in New York City. Toxicol. Commun. 2022, 6, 78–84, doi:10.1080/24734306.2022.2106397.

4. Rodriguez-Osiac, L.; Pinheiro Fernandes, A.C.; Mujica-Coopman, M.-F.; Caro-Moya, P.; Navarro-Rosenblatt, D. A Description of Chilean Foods and Nutrition Health Policies. Rev. Med. Chil. 2021, 149, 1485–1494.

5. Constantin, A.; Cabrera, O.A.; Rios, B.; Barbosa, I.; Ramirez, A.T.; Cina, M.M.; Guzman, S.S. A Human Rights-Based Approach to Non-Communicable Disease: Mandating Front-of-Package Warning Label. Glob. Health 2021, 17, 85, doi:10.1186/s12992-021-00734-z.

6. Duran Aguero, S.; Araneda, J.; Ahumada, D.; Silva Rojas, J.; Buhring, R.; Caichac, A.; Fernandez Salamanca, M.; Villarroel, P.; Fernandez, E.; Pacheco, V.; et al. A Multicenter Study Evaluating the Stages of Change in Foods Consumption with Warning Label among Chilean University Students. BIOMED Res. Int. 2020, 2020, 2317929, doi:10.1155/2020/2317929.

7. Shin, S.; Alqunaibet, A.M.; Alsukait, R.F.; Alruwaily, A.; Alfawaz, R.A.; Algwizani, A.; Herbst, C.H.; Shekar, M.; Finkelstein, E.A. A Randomized Controlled Study to Test Front-of-Package (FOP) Nutrition Label in the Kingdom of Saudi Arabia. NUTRIENTS 2023, 15, 2904, doi:10.3390/nu15132904.

8. Finkelstein, E.A.; Ang, F.J.L.; Doble, B.; Wong, W.H.M.; van Dam, R.M. A Randomized Controlled Trial Evaluating the Relative Effectivenes of the Multiple Traffic Light and Nutri-Score Front of Package Nutrition Label. NUTRIENTS 2019, 11, 2236, doi:10.3390/nu11092236.

9. Ahmed, M.; Oh, A.; Vanderlee, L.; Franco-Arellano, B.; Schermel, A.; Lou, W.; L'Abbe, M. A Randomized Controlled Trial Examining Consumer' Perception and Opinions on Using Different Versions of a foodsFlip© Smartphone Application for Delivery of Nutrition Information. Int. J. Behav. Nutr. Phys. Act. 2020, 17, 22, doi:10.1186/s12966-020-0923-1.

10. Vargas-Meza, J.; Jauregui, A.; Contreras-Manzano, A.; Nieto, C.; Barquera, S. Acceptability and Understanding of Front-of-Package Nutritional Label: An Experimental Study in Mexican Consumer. BMC PUBLIC Health 2019, 19, 1751, doi:10.1186/s12889-019-8108-z.

11. Gupta, A.; Bidla, N.; Joshi, Y.; Prasad, V.; Sachdev, H.S.; Kumar, P. Advertising of Pre-Packaged Foods in India: A Qualitative Analysis. INDIAN Pediatr. 2023, 60, 549–552, doi:10.1007/s13312-023-2933-y.

12. Halim, R.E. Advertising to Kids and Tweens: The Different Effect of Warning Label Attached on the Product Packaging. J. ASIAN FINANCE Econ. Bus. 2019, 6, 193–203, doi:10.13106/jafeb.2019.vol6.no3.193.

13. Taillie, L.S.; Reyes, M.; Arantxa Colchero, M.; Popkin, B.; Corvalan, C. An Evaluation of Chile's Law of Foods Labeling and Advertising on Sugar Sugar Sweetened Beverages Purchases from 2015 to 2017: A before-and-after Study. PLoS Med. 2020, 17, e1003015, doi:10.1371/journal.pmed.1003015.

14. Hamlin, R.; Hamlin, B. An Experimental Comparison of the Impact of "Warning" and "Health Star Rating" FoP Label on Adolescents' Choices of Breakfast Cereals in New Zealand. NUTRIENTS 2020, 12, 1545, doi:10.3390/nu12061545.

15. Saavedra-Garcia, L.; Moscoso-Porras, M.; Diez-Canseco, F. An Experimental Study Evaluating the Influence of Front-of-Package Warning Label on Adolescent's Purchase Intention of Processed Foods Product. Int. J. Environ. Res. Public. Health 2022, 19, 1094, doi:10.3390/ijerph19031094.

16. Duran, R.; Asmitia, E.; Rivera, J.A.; Barquera, S.; Tolentino-Mayo, L. Analysis of Stakeholders' Responses to the Foods Warning Label Regulation in Mexico. Health Res. Policies Syst. 2022, 20, 108, doi:10.1186/s12961-022-00922-2.

17. Ares, G.; Antunez, L.; Cabrera, M.; Thow, A.M. Analysis of the Policies Process for the Implementation of Nutritional Warning Label in Uruguay. PUBLIC Health Nutr. 2021, 24, 5927–5940, doi:10.1017/S1368980021002469.

18. Kanter, R.; Reyes, M.; Vandevijvere, S.; Swinburn, B.; Corvalan, C. Anticipatory Effects of the Implementation of the Chilean Law of Foods Labeling and Advertising on Foods and Beverage Product Reformulation. Obes. Rev. 2019, 20, 129–140, doi:10.1111/obr.12870.

19. David, I.A.; Krutman, L.; Fernandez-Santaella, M.C.; Andrade, J.R.; Andrade, E.B.; Oliveira, L.; Pereira, M.G.; Gomes, F.; Gleiser, S.; Oliveira, J.M.; et al. Appetitive Drives for Ultra-Processed Foods Product and the Ability of Text Warnings to Counteract Consumption Predispositions. PUBLIC Health Nutr. 2018, 21, 543–557, doi:10.1017/S1368980017003263.

20. Miller, C.; Dono, J.; Wakefield, M.; Pettigrew, S.; Coveney, J.; Roder, D.; Durkin, S.; Wittert, G.; Martin, J.; Ettridge, K. Are Australians Ready for Warning Label, Marketing Bans and Sugary Drink Taxes? Two Cross-Sectional Surveys Measuring Support for Policies Responses to Sugar Sugar Sweetened Beverages. BMJ OPEN 2019, 9, e027962, doi:10.1136/bmjopen-2018-027962.

21. Schnettler, B.; Ares, G.; Sepulveda, N.; Bravo, S.; Villalobos, B.; Hueche, C.; Lobos, G. Are Consumer Willing to Pay More for Reformulated Processed Meat Product in the Context of the Implementation of Nutritional Warnings? Case Study with Frankfurters in Chile. MEAT Sci. 2019, 152, 104–108, doi:10.1016/j.meatsci.2019.02.007.

22. Khandpur, N.; Sato, P. de M.; Mais, L.A.; Bortoletto Martins, A.P.; Spinillo, C.G.; Garcia, M.T.; Urquizar Rojas, C.F.; Jaime, P.C. Are Front-of-Package Warning Label More Effective at Communicating Nutrition Information than Traffic-Light Label? A Randomized Controlled Experiment in a Brazilian Sample. NUTRIENTS 2018, 10, 688, doi:10.3390/nu10060688.

23. Nobrega, L.; Ares, G.; Deliza, R. Are Nutritional Warnings More Efficient than Claims in Shaping Consumer' Healthfulnes Perception? Foods Qual. Prefer. 2020, 79, 103749, doi:10.1016/j.foodsqual.2019.103749.

24. Goodman, S.; Vanderlee, L.; Acton, R.B.; Mahamad, S.; Hammond, D. Article The Impact of Front-of-Package Label Design on Consumer Understanding of Nutrient Amounts. NUTRIENTS 2018, 10, 1624, doi:10.3390/nu10111624.

25. Sadrmousavigargari, S.; Cubero Dudinskaya, E.; Mandolesi, S.; Naspetti, S.; Mojaverian, S.M.; Zanoli, R. Assessing Consumer Willingness to Pay for Nutritional Information Using a Dietary App. NUTRIENTS 2022, 14, 5023, doi:10.3390/nu14235023.

26. Packer, J.; Russell, S.J.; Ridout, D.; Hope, S.; Conolly, A.; Jessop, C.; Robinson, O.J.; Stoffel, S.T.; Viner, R.M.; Croker, H. Assessing the Effectivenes of Front of Pack Label: Findings from an Online Randomised-Controlled Experiment in a Representative British Sample. NUTRIENTS 2021, 13, 900, doi:10.3390/nu13030900.

27. Melendez-Illanes, L.; Olivares Cortes, S.; Saez-Carrillo, K.; Zapata Fuentes, D.; Munoz Reyes, S.; Granfeldt Molina, G. attitudes of mothers of preschools in the implementation of the nutritional labeling law in Chile. Arch. Latinoam. Nutr. 2019, 69, 165-+.

28. Mandal, S.K.; Mitra, A.; Alok, Y.; Gupta, S.; Majumdar, A. Awarenes and Perception Regarding Taxation and Health Warnings Related to Sugar Sugar Sweetened Beverages and the Factors Associated with These among Visitors of a

General Out-Patient Clinic in Bhopal, India. J. Fam. Med. Prim. CARE 2020, 9, 2350–2358, doi:10.4103/jfmpc.jfmpc_226_20.

29. Hammond, D.; Acton, R.B.; Rynard, V.L.; White, C.M.; Vanderlee, L.; Bhawra, J.; Reyes, M.; Jauregui, A.; Adams, J.; Roberto, C.A.; et al. Awarenes, Use and Understanding of Nutrition Label among Children and Youth from Six Countries: Findings from the 2019-2020 International Foods Policies Study. Int. J. Behav. Nutr. Phys. Act. 2023, 20, 55, doi:10.1186/s12966-023-01455-9.

30. Lytton, T.D. Banning Front-of-Package Foods Label: First Amendment Constraints on Public Health Policies. PUBLIC Health Nutr. 2011, 14, 1123–1126, doi:10.1017/S1368980010002843.

31. Mais, L.A.; Borges, C.A.; Khandpur, N.; Duran, A.C.; Martins, A.P.B. Brazil's Nutrition Labeling Regulation: Challenges Ahead on the Path to Guaranteeing Consumer's Right to Adequate Information. Front. Nutr. 2022, 9, 921519, doi:10.3389/fnut.2022.921519.

32. Felipetto, N.; Scheffer, P.A.; de Mattos Margutti, K.M.; Silveira, J.T.; Marques, C.T.; Storck, C.R.; de Oliveira, V.R.; Helbig, E.; Ginani, V.C.; de Freitas Saccol, A.L. Brazilian Consumer' Perception towards Foods Labeling Model Accompanying Self-Service Foods. foods 2022, 11, 838, doi:10.3390/foods11060838.

33. Andreeva, V.A.; Egnell, M.; Handjieva-Darlenska, T.; Talati, Z.; Touvier, M.; Galan, P.; Hercberg, S.; Pettigrew, S.; Julia, C. Bulgarian Consumer' Objective Understanding of Front-of-Package Nutrition Label: A Comparative, Randomized Study. Arch. PUBLIC Health 2020, 78, 35, doi:10.1186/s13690-020-00416-z.

34. Reyes-Sanchez, F.; Basto-Abreu, A.; Torres-Alvarez, R.; Carnalla-Cortes, M.; Reyes-Garcia, A.; Swinburn, B.; Meza, R.; Rivera, J.A.; Popkin, B.; Barientos-Gutierrez, T. Caloric Reductions Needed to Achieve Obesity Goals in Mexico for 2030 and 2040: A Modeling Study. PLoS Med. 2023, 20, e1004248, doi:10.1371/journal.pmed.1004248.

35. Lima, M.; de Alcantara, M.; Martins, I.B.A.; Ares, G.; Deliza, R. Can Front-of-Package Nutrition Labeling Influence Children's Emotional Associations with Unhealth Foods Product? An Experiment Using Emoji. Foods Res. Int. 2019, 120, 217–225, doi:10.1016/j.foodsres.2019.02.027.

36. Machin, L.; Arrua, A.; Gimenez, A.; Curutchet, M.R.; Martinez, J.; Ares, G. Can Nutritional Information Modify Purchase of Ultra-Processed Foods? Results from a Simulated Online Shopping Experiment. PUBLIC Health Nutr. 2018, 21, 49–57, doi:10.1017/S1368980017001185.

37. Foster, N.D.; Constantin, A.; Lake, S.J. CARICOM'S EFFORTS TO TACKLE UNhealth diet: A CAUTIONARY TALE FOR LATIN AMERICA. Rev. Estud. Inst.-J. INSTITUTIONAL Stud. 2022, 8, 254–279, doi:10.21783/rei.v8i2.663.

38. Fretes, G.; Corvalan, C.; Reyes, M.; Taillie, L.S.; Economos, C.D.; Wilson, N.L.W.; Cash, S.B. Changes in Children's and Adolescents' Dietary Intake after the Implementation of Chile's Law of Foods Labeling, Advertising and Sales in Schools: A Longitudinal Study. Int. J. Behav. Nutr. Phys. Act. 2023, 20, 40, doi:10.1186/s12966-023-01445-x.

39. Taillie, L.S.; Bercholz, M.; Popkin, B.; Reyes, M.; Arantxa Colchero, M.; Corvalan, C. Changes in Foods Purchases after the Chilean Policies on Foods Labelling, Marketing, and Sales in Schools: A before and after Study. LANCET Planet. Health 2021, 5, E526–E533.

40. Quintiliano Scarpelli, D.; Pinheiro Fernandes, A.C.; Rodriguez-Osiac, L.; Pizarro Quevedo, T. Changes in Nutrient Declaration after the Foods Labeling and Advertising Law in Chile: A Longitudinal Approach. NUTRIENTS 2020, 12, 2371, doi:10.3390/nu12082371.

41. Reyes, M.; Taillie, L.S.; Popkin, B.; Kanter, R.; Vandevijvere, S.; Corvalan, C. Changes in the Amount of Nutrient of Packaged Foods and Beverages after the Initial Implementation of the Chilean Law of Foods Labelling and Advertising: A Nonexperimental Prospective Study. PLoS Med. 2020, 17, e1003220, doi:10.1371/journal.pmed.1003220.

42. Bragge, P.; Waddell, A.; Kellner, P.; Delafosse, V.; Marten, R.; Nordstrom, A.; Demaio, S. Characteristics of Successful Government-Led Intervention to Support Healthier Populations: A Starting Portfolio of Positive Outlier Examples. BMJ Glob. Health 2023, 8, e011683, doi:10.1136/bmjgh-2023-011683.

43. Fretes, G.; Sepulveda, A.; Corvalan, C.; Cash, S.B. Children's Perception about Environmental Sustainability, Foods, and Nutrition in Chile: A Qualitative Study. Int. J. Environ. Res. Public. Health 2021, 18, 9679, doi:10.3390/ijerph18189679.

44. Campbell, M. Chile: Front-of-Package Warning Label and Foods Marketing. J. Law. Med. Ethics 2022, 50, 298–303, doi:10.1017/jme.2022.55.

45. Khandpur, N.; Mais, L.A.; Sato, P. de M.; Bortoletto Martins, A.P.; Spinillo, C.G.; Urquizar Rojas, C.F.; Garcia, M.T.; Jaime, P.C. Choosing a Front-of-Package Warning Label for Brazil: A Randomized, Controlled Comparison of Three Different Label Designs. Foods Res. Int. 2019, 121, 854–861, doi:10.1016/j.foodsres.2019.01.008.

46. Ares, G.; Varela, F.; Machin, L.; Antunez, L.; Gimenez, A.; Curutchet, M.R.; Aschemann-Witzel, J. Comparative Performance of Three Interpretative Front-of-Package Nutrition Labelling Scheme: Insights for Policies Making. Foods Qual. Prefer. 2018, 68, 215–225, doi:10.1016/j.foodsqual.2018.03.007.

47. Egnell, M.; Galan, P.; Farpour-Lambert, N.J.; Talati, Z.; Pettigrew, S.; Hercberg, S.; Julia, C. Compared to Other Front-of-Package Nutrition Label, the Nutri-Score Emerged as the Most Efficient to Inform Swiss Consumer on the Nutritional Quality of Foods Product. PLoS ONE 2020, 15, e0228179, doi:10.1371/journal.pone.0228179.

48. Borges, C.A.; Khandpur, N.; Neri, D.; Duran, A.C. Comparing Latin American Nutrient Profile Model Using Data from Packaged Foods with Child-Directed Marketing within the Brazilian Foods Supply. Front. Nutr. 2022, 9, 920710, doi:10.3389/fnut.2022.920710.

49. Aguenaou, H.; El Ammari, L.; Bigdeli, M.; El Hajjab, A.; Lahmam, H.; Labzizi, S.; Gamih, H.; Talouizte, A.; Serbouti, C.; El Kari, K.; et al. Comparison of Appropriateness of Nutri-Score and Other Front-of-Package Nutrition Label across a Group of Moroccan Consumer: Awarenes, Understanding and Foods Choices. Arch. PUBLIC Health 2021, 79, 71, doi:10.1186/s13690-021-00595-3.

50. Egnell, M.; Talati, Z.; Pettigrew, S.; Galan, P.; Hercberg, S.; Julia, C. Comparison of Front-of-Package Label to Help German Consumer Understand the Nutritional Quality of Foods Product. ERNAHRUNGS Umsch. 2019, 66, 76–84, doi:10.4455/eu.2019.020.

51. Quitral, V.; Arteaga, J.; Rivera, M.; Galleguillos, J.; Valdes, I. Comparison of sugar and non- caloric sweetener content in beverages before and after implementing Chilean law 20.606. Rev. Chil. Nutr. 2019, 46, 245–253, doi:10.4067/S0717-75182019000300245.

52. Silva, A.R.C.S.; Ni Mhurchu, C.; Anastacio, L.R. Comparison of Two Front-of-Package Nutrition Label for Brazilian Consumer Using a Smartphone App in a Real-World Grocery Store: A Pilot Randomized Controlled Study. Front. Nutr. 2022, 9, 898021, doi:10.3389/fnut.2022.898021.

53. Adams, J.M.; Hart, W.; Gilmer, D.O.; Lloyd-Richardson, E.E.; Burton, K.A. Concrete Images of the Sugar Content in Sugar Sugar Sweetened Beverages Reduces Attraction to and Selection of These Beverages. APPETITE 2014, 83, 10–18, doi:10.1016/j.appet.2014.07.027.

54. Duran, A.C.; Ricardo, C.Z.; Mais, L.A.; Bortoletto Martins, A.P.; Taillie, L.S. Conflicting Messages on Foods and Beverage Packages: Front-of-Package

Nutritional Labeling, Health and Nutrition Claims in Brazil. NUTRIENTS 2019, 11, 2967, doi:10.3390/nu11122967.

55. Claro, R.M.; Linders, H.; Ricardo, C.Z.; Legetic, B.; Campbell, N. Consumer Attitudes, Knowledge, and Behavior Related to Salt Consumption in Sentinel Countries of the Americas. Rev. Panam. SALUD PUBLICA-PAN Am. J. PUBLIC Health 2012, 32, 265–273, doi:10.1590/S1020-49892012001000004.

56. Acton, R.B.; Vanderlee, L.; Roberto, C.A.; Hammond, D. Consumer Perception of Specific Design Characteristics for Front-of-Package Nutrition Label. Health Educ. Res. 2018, 33, 167–174, doi:10.1093/her/cyy006.

57. Antunez, L.; Curutchet, M.R.; Moratorio, X.; Gimenez, A.; Ares, G. Consumer Perspective on the Inclusion of Nutritional Warnings in Retail Foods Establishments. Rev. Chil. Nutr. 2022, 49, 43–52, doi:10.4067/S0717-75182022000100043.

58. Ghosh, R.K.; Sanghvi, R.; Sahay, A. Consumer Preference for Nutrition Front-of-Package-Label Formats in India: Evidence from a Large-Scale Experimental Survey. Foods Qual. Prefer. 2023, 111, 104993, doi:10.1016/j.foodsqual.2023.104993.

59. Ouyang, Y.; Sharma, A. Consumer-Citizen Willingness to Pay for Health Eating Messages. Int. J. Contemp. Hosp. Manag. 2019, 31, 890–909, doi:10.1108/IJCHM-08-2017-0490.

60. Vandevijvere, S.; Vermote, M.; Egnell, M.; Galan, P.; Talati, Z.; Pettigrew, S.; Hercberg, S.; Julia, C. Consumer' Foods Choices, Understanding and Perception in Response to Different Front-of-Package Nutrition Labelling System in Belgium: Results from an Online Experimental Study. Arch. PUBLIC Health 2020, 78, 30, doi:10.1186/s13690-020-00404-3.

61. Sato, P. de M.; Mais, L.A.; Khandpur, N.; Ulian, M.D.; Bortoletto Martins, A.P.; Garcia, M.T.; Spinillo, C.G.; Urquizar Rojas, C.F.; Jaime, P.C.; Scagliusi, F.B. Consumer' Opinions on Warning Label on Foods Packages: A Qualitative Study in Brazil. PLoS ONE 2019, 14, e0218813, doi:10.1371/journal.pone.0218813.

62. Bhattacharya, S.; Bera, O.P.; Shah, V. Consumer' Perception About Front of Package Foods Label (FOPL) in India: A Survey of 14 States. Front. PUBLIC Health 2022, 10, 936802, doi:10.3389/fpubh.2022.936802.

63. Talati, Z.; Egnell, M.; Hercberg, S.; Julia, C.; Pettigrew, S. Consumer' Perception of Five Front-of-Package Nutrition Label: An Experimental Study Across 12 Countries. NUTRIENTS 2019, 11, 1934, doi:10.3390/nu11081934.

64. Zhang, X.; Ouyang, Y.; Yin, X.; Bai, J.; Zhang, R.; Zhang, J.; Wang, Q.; Wang, H. Consumer' Perception of the Design of Front-of-Package Warning Label-A Qualitative Study in China. NUTRIENTS 2023, 15, 415, doi:10.3390/nu15020415.

65. Scapin, T.; Fernandes, A.C.; Shahid, M.; Pettigrew, S.; Khandpur, N.; Bernardo, G.L.; Uggioni, P.L.; da Costa Proenca, R.P. Consumer' Response to Sugar Label Formats in Packaged Foods: A Multi-Methods Study in Brazil. Front. Nutr. 2022, 9, 896784, doi:10.3389/fnut.2022.896784.

66. Egnell, M.; Talati, Z.; Gombaud, M.; Galan, P.; Hercberg, S.; Pettigrew, S.; Julia, C. Consumer' Responses to Front-of-Package Nutrition Labelling: Results from a Sample from The Netherlands. NUTRIENTS 2019, 11, 1817, doi:10.3390/nu11081817.

67. Hashem, K.M.; He, F.J.; MacGregor, G.A. Cross-Sectional Surveys of the Amount of Sugar, Energy and Caffeine in Sugar-Sweetened Drink Marketed and Consumed as Energy Drink in the UK between 2015 and 2017: Monitoring Reformulation Progress. BMJ OPEN 2017, 7, e018136, doi:10.1136/bmjopen-2017-018136.

68. Jauregui, A.; Pacheco-Miranda, S.; Ayvar-Gama, Y.; Alejandro-Torres, N.Z.; Cuno, A.; Fiorella Espinosa-de Candido, A.; Martinez-Cruz, M.I.; Bonvecchio-Arenas, A.; Barquera, S. Design of a communication strategy to promote the use of warning label among Mexican children and adolescents. SALUD PUBLICA Mex. 2023, 65, 70–81, doi:10.21149/14120.

69. Carrero, I.; Valor, C.; Diaz, E.; Labajo, V. Designed to Be Noticed: A Reconceptualization of Carbon Foods Label as Warning Label. SusTAINABILITY 2021, 13, 1581, doi:10.3390/su13031581.

70. Seyedhamzeh, S.; Nedjat, S.; Shakibazadeh, E.; Doustmohammadian, A.; Hosseini, H.; Kazemi, A.; Azizolahi, N.; Chamary, M.; Clark, C.C.T.; Motlagh, A.D. Designing a New Physical Activity Calorie Equivalent Foods Label and Comparing Its Effect on Caloric Choices to That of the Traffic Light Label among Mothers: A Mixed-Method Study. Front. PUBLIC Health 2023, 11, 1280532, doi:10.3389/fpubh.2023.1280532.

71. Taillie, L.S.; Hall, M.; Gomez, L.F.; Higgins, I.C.A.; Bercholz, M.; Murukutla, N.; Mora-Plazas, M. Designing an Effective Front-of-Package Warning Label for Foods and Drink High in Added Sugar, Sodium, or Saturated Fat in Colombia: An Online Experiment. NUTRIENTS 2020, 12, 3124, doi:10.3390/nu12103124.

72. Falbe, J.; Montuclard, A.; Engelman, A.; Adler, S.; Roesler, A. Developing Sugar Sugar Sweetened Beverages Warning Label for Young Adults. PUBLIC Health Nutr. 2021, 24, 4765–4775, doi:10.1017/S1368980021002287.

73. Gillon-Keren, M.; Kaufman-Shriqui, V.; Goldsmith, R.; Safra, C.; Shai, I.; Fayman, G.; Berry, E.; Tirosh, A.; Dicker, D.; Froy, O.; et al. Development of Criteria for a Positive Front-of-Package Foods Labeling: The Israeli Case. NUTRIENTS 2020, 12, 1875, doi:10.3390/nu12061875.

74. Reyes, M.; Garmendia, M.L.; Olivares Cortes, S.; Aqueveque, C.; Zacarias, I.; Corvalan, C. Development of the Chilean Front-of-Package Foods Warning Label. BMC PUBLIC Health 2019, 19, 906, doi:10.1186/s12889-019-7118-1.

75. Valenzuela, A.; Zambrano, L.; Velasquez, R.; Groff, C.; Apablaza, T.; Riffo, C.; Moldenhauer, S.; Brisso, P.; Leonario-Rodriguez, M. Discrepancy between Foods Classification System: Evaluation of Nutri-Score, NOVA Classification and Chilean Front-of-Package Foods Warning Label. Int. J. Environ. Res. Public. Health 2022, 19, 14631, doi:10.3390/ijerph192214631.

76. Acton, R.B.; Hammond, D. Do Consumer Think Front-of-Package “High in” Warnings Are Harsh or Reduce Their Control? A Test of Foods Industry Concerns. OBESITY 2018, 26, 1687–1691, doi:10.1002/oby.22311.

77. Annunziata, A.; Pomarici, E.; Vecchio, R.; Mariani, A. Do Consumer Want More Nutritional and Health Information on Wine Label? Insights from the EU and usA. NUTRIENTS 2016, 8, 416, doi:10.3390/nu8070416.

78. Acton, R.B.; Hammond, D. Do Manufacturer “nutrient Claims” Influence the Efficacy of Mandated Front-of-Package Label? PUBLIC Health Nutr. 2018, 21, 3354–3359, doi:10.1017/S1368980018002550.

79. Machin, L.; Curutchet, M.R.; Gimenez, A.; Aschemann-Witzel, J.; Ares, G. Do Nutritional Warnings Do Their Work? Results from a Choices Experiment Involving Snack Product. Foods Qual. Prefer. 2019, 77, 159–165, doi:10.1016/j.foodsqual.2019.05.012.

80. Gugliucci, V.; Machin, L.; Curutchet, M.R.; Ares, G. Do Nutritional Warnings Encourage Healthier Choices on Foods Ordering Websites? An Exploratory Experimental Study in Uruguay. PUBLIC Health Nutr. 2021, 24, 3547–3551, doi:10.1017/S1368980021001026.

81. Mais, L.A.; Mialon, M.; Hassan, B.K.; Darre Peres, J.M.; dos Santos, M.G.; Bortoletto Martins, A.P.; Coutinho, J.G.; Paes de Carvalho, C.M. Do They Really Support “Your Freedom of Choices”? FoPNL and the Foods Industry in Brazil. Front. Nutr. 2023, 9, 921498, doi:10.3389/fnut.2022.921498.

82. Machin, L.; Aschemann-Witzel, J.; Curutchet, M.R.; Gimenez, A.; Ares, G. Does Front-of-Package Nutrition Information Improve Consumer Ability to Make Healthful Choices? Performance of Warnings and the Traffic Light System in a

Simulated Shopping Experiment. APPETITE 2018, 121, 55–62, doi:10.1016/j.appet.2017.10.037.

83. Vedha, V.P.K.; Mohanty, V.R.; Balappanavar, A.Y.; Kapoor, S.; Gupta, V. Does Pictorial and Textual Health Warning Label on Sugar-Sweetened Beverages Effect Consumer Consumption: An Exploratory, Cross-Sectional Study. J. INDIAN Assoc. PUBLIC Health Dent. 2023, 21, 359–364, doi:10.4103/jiaphd.jiaphd_129_22.

84. Caballero, S.; Moenne-Loccoz, C.; Delgado, M.; Luarte, L.; Jimenez, Y.; Galgani, J.E.; Perez-Leighton, C.E.E. Eating Contexts Determine the Efficacy of Nutrient Warning Label to Promote Health Foods Choices. Front. Nutr. 2023, 9, 1026623, doi:10.3389/fnut.2022.1026623.

85. Mendoza, D.J.; Mejia, C.R. Effect of a health education program on students in the Peruvian highlands during COVID-19. Nutr. Clin. Diet. Hosp. 2021, 41, 141–149, doi:10.12873/413mendoza.

86. Vandevijvere, S.; Vanderlee, L. Effect of Formulation, Labelling, and Taxation Policies on the Nutritional Quality of the Foods Supply. Curr. Nutr. Rep. 2019, 8, 240–249, doi:10.1007/s13668-019-00289-x.

87. Musicus, A.A.; Roberto, C.A.; Moran, A.J.; Sorscher, S.; Greenthal, E.; Rimm, E.B. Effect of Front-of-Package Information, Fruit Imagery, and High-Added Sugar Warning Label on Parent Beverage Choices for Children A Randomized Clinical Trial. JAMA Netw. OPEN 2022, 5, e2236384, doi:10.1001/jamanetworkopen.2022.36384.

88. Adasme-Berrios, C.; Aliaga-Ortega, L.; Schnettler, B.; Parada, M.; Andaur, Y.; Carreno, C.; Lobos, G.; Jara-Rojas, R.; Valdes, R. Effect of Warning Label on Consumer Motivation and Intention to Avoid Consuming Processed Foods. NUTRIENTS 2022, 14, 1547, doi:10.3390/nu14081547.

89. Fialon, M.; Egnell, M.; Talati, Z.; Galan, P.; Dreano-Trecant, L.; Touvier, M.; Pettigrew, S.; Hercberg, S.; Julia, C. Effectivenes of Different Front-of-Package Nutrition Label among Italian Consumer: Results from an Online Randomized Controlled Trial. NUTRIENTS 2020, 12, 2307, doi:10.3390/nu12082307.

90. Grummon, A.H.; Gibson, L.A.; Musicus, A.A.; Stephens-Shields, A.J.; Hua, S.V.; Roberto, C.A. Effects of 4 Interpretive Front-of-Package Labeling System on Hypothetical Beverage and Snack Selections A Randomized Clinical Trial. JAMA Netw. OPEN 2023, 6, e2333515, doi:10.1001/jamanetworkopen.2023.33515.

91. Neal, B.; Crino, M.; Dunford, E.; Gao, A.; Greenland, R.; Li, N.; Ngai, J.; Ni Mhurchu, C.; Pettigrew, S.; Sacks, G.; et al. Effects of Different Types of Front-of-Package Labelling Information on the Healthiness of Foods PurchasesA Randomised Controlled Trial. NUTRIENTS 2017, 9, 1284, doi:10.3390/nu9121284.

92. Wang, C.-Y.; Hsu, C.-J.; Cai, D. Effects of Foods Nutrition Label on the Health Awarenes of School-Age Children. BMC PUBLIC Health 2022, 22, 1249, doi:10.1186/s12889-022-13613-y.

93. White-Barrow, V.; Gomes, F.; Eyre, S.; Ares, G.; Morris, A.; Caines, D.; Finlay, D. Effects of Front-of-Package Nutrition Labelling System on Understanding and Purchase Intention in Jamaica: Results from a Multiarm Randomised Controlled Trial. BMJ OPEN 2023, 13, e065620, doi:10.1136/bmjopen-2022-065620.

94. Liem, D.G.; Aydin, N.T.; Zandstra, E.H. Effects of Health Label on Expected and Actual Taste Perception of Soup. Foods Qual. Prefer. 2012, 25, 192–197, doi:10.1016/j.foodsqual.2012.02.015.

95. Chiou, W.-B.; Yeh, L.-T.; Chang, M.-H. EFFECTS OF HEALTH-RELATED CLAIMS ON THE DIFFERENTIAL THRESHOLD OF Consumer' SWEETNESS SENSATION. J. Sens. Stud. 2009, 24, 621–633, doi:10.1111/j.1745-459X.2009.00230.x.

96. Mehlhose, C.; Risius, A. Effects of Immediate and Distant Health Consequences: Different Types of Health Warning Messages on Sweets Affect the Purchase Probability. BMC PUBLIC Health 2023, 23, 1892, doi:10.1186/s12889-023-16760-y.

97. Bollard, T.; Maubach, N.; Walker, N.; Mhurchu, C.N. Effects of Plain Packaging, Warning Label, and Taxes on Young People's Predicted Sugar Sugar Sweetened Beverages Preference: An Experimental Study. Int. J. Behav. Nutr. Phys. Act. 2016, 13, 95, doi:10.1186/s12966-016-0421-7.

98. Bushman, B.J. Effects of Warning and Information Label on Consumption of Full-Fat, Reduced-Fat, and No-Fat Product. J. Appl. Psychol. 1998, 83, 97–101, doi:10.1037/0021-9010.83.1.97.

99. Mansfield, E.D.; Ibanez, D.; Chen, F.; Chen, E.; de Grandpre, E. Efficacy of "High in" Nutrient Specific Front of Package Label-A Retail Experiment with Canadians of Varying Health Literacy Levels. NUTRIENTS 2020, 12, 3199, doi:10.3390/nu12103199.

100. Castronuovo, L.; Tiscornia, M.V.; Guarnieri, L.; Martins, E.; Gomes, F.; Allemandi, L. Efficacy of Different Front-of-Package Labeling System in Changing Purchase Intention and Product Healthfulnes Perception for Foods Product in Argentina. Rev. Panam. SALUD PUBLICA-PAN Am. J. PUBLIC Health 2022, 46, e137, doi:10.26633/RPSP.2022.137.

101. Kroker-Lobos, M.F.; Morales-Juarez, A.; Perez, W.; Kanda, T.; Gomes, F.; Ramirez-Zea, M.; Siu-Bermudez, C. Efficacy of Front-of-Package Warning Label System versus Guideline for Daily Amount on Healthfulnes Perception, Purchase

Intention and Objective Understanding of Nutrient Content of Foods Product in Guatemala: A Cross-over Cluster Randomized Controlled Experiment. Arch. PUBLIC Health 2023, 81, 108, doi:10.1186/s13690-023-01124-0.

102. Barahona, N.; Otero, C.; Otero, S. Equilibrium Effects of Foods Labeling Policies. ECONOMETRICA 2023, 91, 839–868, doi:10.3982/ECTA19603.

103. Cruz-Casarrubias, C.; Tolentino-Mayo, L.; Vandevijvere, S.; Barquera, S. Estimated Effects of the Implementation of the Mexican Warning Label Regulation on the Use of Health and Nutrition Claims on Packaged Foods. Int. J. Behav. Nutr. Phys. Act. 2021, 18, 76, doi:10.1186/s12966-021-01148-1.

104. Flexner, N.; Ng, A.P.; Ahmed, M.; Khandpur, N.; Acton, R.B.; Lee, J.J.J.; L'Abbe, M. Estimating the Dietary and Health Impact of Implementing Front-of-Package Nutrition Labeling in Canada: A Macrosimulation Modeling Study. Front. Nutr. 2023, 10, 1098231, doi:10.3389/fnut.2023.1098231.

105. Grummon, A.H.; Hall, M.; Block, J.P.; Bleich, S.N.; Rimm, E.B.; Taillie, L.S.; Barnhill, A. Ethical Considerations for Foods and Beverage Warnings. Physiol. Behav. 2020, 222, 112930, doi:10.1016/j.physbeh.2020.112930.

106. Alaniz-Salinas, N.; Castillo-Montes, M. Evaluation of the front-of-package warning label of the Chilean foods Law by adults responsible for schoolchildren in the districts of La Serena and Coquimbo. Rev. Chil. Nutr. 2020, 47, 738–749, doi:10.4067/S0717-75182020000500738.

107. Contreras-Manzano, A.; Cruz-Casarrubias, C.; Munguia, A.; Jauregui, A.; Vargas-Meza, J.; Nieto, C.; Tolentino-Mayo, L.; Barquera, S. Evaluation of the Mexican Warning Label Nutrient Profile on Foods Product Marketed in Mexico in 2016 and 2017: A Cross-Sectional Analysis. PLoS Med. 2022, 19, e1003968, doi:10.1371/journal.pmed.1003968.

108. Santana, A.H.; Andrade, S.W.B.; Aleman, D.R.; Enriquez, J.P.; Di Iorio, A.B. Evaluation of the Nutritional Quality of Processed Foods in Honduras: Comparison of Three Nutrient Profiles. Int. J. Environ. Res. Public. Health 2020, 17, 7060, doi:10.3390/ijerph17197060.

109. Paraje, G.; de Oca, D.M.; Corvalan, C.; Popkin, B. Evolution of Foods and Beverage Price after the Front-of-Package Labelling Regulations in Chile. BMJ Glob. Health 2023, 8, e011312, doi:10.1136/bmjgh-2022-011312.

110. Pereira, R.C.; de Angelis-Pereira, M.C.; Souza Carneiro, J. de D. Exploring Claims and Marketing Techniques in Brazilian Foods Label. Br. Foods J. 2019, 121, 1550–1564, doi:10.1108/BFJ-08-2018-0516.

111. Riesenberg, D.; Peeters, A.; Backholer, K.; Martin, J.; Mhurchu, C.N.; Blake, M.R. Exploring the Effects of Added Sugar Label on Foods Purchasing Behaviour in Australian Parents: An Online Randomised Controlled Trial. PLoS ONE 2022, 17, e0271435, doi:10.1371/journal.pone.0271435.

112. Adasme-Berrios, C.; Carreno, C.; Aliaga-Ortega, L.; Schnettler, B.; Lobos, G. Factors That Determine Choices of Processed Foods among University Students in the Context of Nutritional Warning Label. Rev. Chil. Nutr. 2022, 49, 451–458, doi:10.4067/S0717-75182022000500451.

113. Talati, Z.; Egnell, M.; Hercberg, S.; Julia, C.; Pettigrew, S. Foods Choices Under Five Front-of-Package Nutrition Label Conditions: An Experimental Study Across 12 Countries. Am. J. PUBLIC Health 2019, 109, 1770–1775, doi:10.2105/AJPH.2019.305319.

114. Ares, G.; Bove, I.; Diaz, R.; Moratorio, X.; Benia, W.; Gomes, F. foods industry arguments against front-of-package nutrition label in Uruguay. Rev. Panam. SALUD PUBLICA-PAN Am. J. PUBLIC Health 2020, 44, 44, doi:10.26633/RPSP.2020.20.

115. Tomaz, L.A.; Pereira, C.G.; Mascarenhas Braga, L.V.; Senna Prates, S.M.; Carolino Sales Silva, A.R.; da Costa Soares, A.P.; de Faria, N.C.; Anastacio, L.R. From the Most to the Least Flexible Nutritional Profile: Classification of Foods Marketed in Brazil According to the Brazilian and Mexican Model. Front. Nutr. 2022, 9, 919582, doi:10.3389/fnut.2022.919582.

116. Singh, S.K.; Taillie, L.S.; Gupta, A.; Bercholz, M.; Popkin, B.; Murukutla, N. Front-of-Package Label on Unhealth Packaged Foods in India: Evidence from a Randomized Field Experiment. NUTRIENTS 2022, 14, 3128, doi:10.3390/nu14153128.

117. Vargas-Meza, J.; Jauregui, A.; Pacheco-Miranda, S.; Contreras-Manzano, A.; Barquera, S. Front-of-Package Nutritional Label: Understanding by Low- and Middle-Income Mexican Consumer. PLoS ONE 2019, 14, e0225268, doi:10.1371/journal.pone.0225268.

118. de Alcantara, M.; Ares, G.; Leme de Castro, I.P.; Deliza, R. Gain vs. Loss-Framing for Reducing Sugar Consumption: Insights from a Choices Experiment with Six Product Categories. Foods Res. Int. 2020, 136, 109458, doi:10.1016/j.foodsres.2020.109458.

119. Fernan, C.; Schuldt, J.P.; Niederdeppe, J. Health Halo Effects from Product Titles and Nutrient Content in the Context of “Protein” Bars. Health Commun. 2018, 33, 1425–1433, doi:10.1080/10410236.2017.1358240.

120. Todd, M.; Guetterman, T.; Volschenk, J.; Kidd, M.; Joubert, E. Health or Not Health? A Mixed-Methods Approach to Evaluate Front-of-Package Nutrition Label

as a Tool to Guide Consumer. NUTRIENTS 2022, 14, 2801, doi:10.3390/nu14142801.

121. Moran, A.J.; Roberto, C.A. Health Warning Label Correct Parents' Misperception About Sugary Drink Options. Am. J. Prev. Med. 2018, 55, E19–E27, doi:10.1016/j.amepre.2018.04.018.

122. Grummon, A.H.; Brewer, N.T. Health Warnings and Beverage Purchase Behavior: Mediators of Impact. Ann. Behav. Med. 2020, 54, 691–702, doi:10.1093/abm/kaaa011.

123. Lacanilao, R.D.; Cash, S.B.; Adamowicz, W.L. Heterogeneous Consumer Responses to Snack Foods Taxes and Warning Label. J. Consum. Aff. 2011, 45, 108–122, doi:10.1111/j.1745-6606.2010.01194.x.

124. Ares, G.; Antunez, L.; Gugliucci, V.; Curutchet, M.R.; Galicia, L.; Moratorio, X.; Gimenez, A.; Bove, I. How Do Consumer Characteristics Influence Responses to Nutritional Warnings? Rev. Chil. Nutr. 2021, 48, 578–585, doi:10.4067/S0717-75182021000400578.

125. Schnettler, B.; Ares, G.; Sepulveda, N.; Bravo, S.; Villalobos, B.; Hueche, C.; Adasme-Berrios, C. How Do Consumer Perceive Reformulated Foods after the Implementation of Nutritional Warnings? Case Study with Frankfurters in Chile. Foods Qual. Prefer. 2019, 74, 179–188, doi:10.1016/j.foodsqual.2019.01.021.

126. Deliza, R.; de Alcantara, M.; Pereira, R.; Ares, G. How Do Different Warning Signs Compare with the Guideline Daily Amount and Traffic-Light? Foods Qual. Prefer. 2020, 80, 103821, doi:10.1016/j.foodsqual.2019.103821.

127. Lima, M.; Ares, G.; Deliza, R. How Do Front of Pack Nutrition Label Affect Healthfulnes Perception of Foods Targeted at Children? Insights from Brazilian Children and Parents. Foods Qual. Prefer. 2018, 64, 111–119, doi:10.1016/j.foodsqual.2017.10.003.

128. de Alcantara, M.; Ares, G.; Deliza, R. How Do Nutritional Warnings Work on Commercial Product? Results From a Hypothetical Choices Experiment. Front. Nutr. 2022, 9, 921515, doi:10.3389/fnut.2022.921515.

129. Chien-Huang, L.; Hung-Chou, L. How Health Information Affects College Students' Inclination toward Variety-Seeking Tendency. Scand. J. Psychol. 2010, 51, 503–508, doi:10.1111/j.1467-9450.2010.00815.x.

130. Castagnoli, J. de L.; dos Santos, E.F.; Novello, D. How Interdisciplinary Intervention Can Improve the Educational Process of Children Regarding the Nutritional Labeling of Foods. foods 2023, 12, 4290, doi:10.3390/foods12234290.

131. Hall, M.; Grummon, A.H.; Queen, T.; Lazard, A.J.; Higgins, I.C.A.; Richter, A.P.C.; Taillie, L.S. How Pictorial Warnings Change Parents' Purchases of Sugar-Sweetened Beverage for Their Children: Mechanisms of Impact. Int. J. Behav. Nutr. Phys. Act. 2023, 20, 76, doi:10.1186/s12966-023-01469-3.

132. Grummon, A.H.; Hall, M.; Taillie, L.S.; Brewer, N.T. How Should Sugar Sugar Sweetened Beverages Health Warnings Be Designed? A Randomized Experiment. Prev. Med. 2019, 121, 158–166, doi:10.1016/j.ypmed.2019.02.010.

133. Villaverde, P.; Tolentino-Mayo, L.; Cruz-Casarrubias, C.; Salgado, J.C.; Aburto, T.C.; Barquera, S. Hypothetical Impact of the Mexican Front-of-Package Labeling on Intake of Critical Nutrients and Energy. J. Health Popul. Nutr. 2023, 42, 124, doi:10.1186/s41043-023-00462-7.

134. Mialon, M.; Gaitan Charry, D.A.; Cediel, G.; Crosbie, E.; Scagliusi, F.B.; Perez Tamayo, E.M. "I Had Never Seen so Many Lobbyists": Foods Industry Political Practices during the Development of a New Nutrition Front-of-Package Labelling System in Colombia. PUBLIC Health Nutr. 2021, 24, 2737–2745, doi:10.1017/S1368980020002268.

135. Ares, G.; Antunez, L.; Curutchet, M.R.; Galicia, L.; Moratorio, X.; Gimenez, A.; Bove, I. Immediate Effects of the Implementation of Nutritional Warnings in Uruguay: Awarenes, Self-Reported Use and Increased Understanding. PUBLIC Health Nutr. 2021, 24, 364–375, doi:10.1017/S1368980020002517.

136. Song, J.; Brown, M.K.; Tan, M.; MacGregor, G.A.; Webster, J.J.; Campbell, N.; Trieu, K.; Ni Mhurchu, C.; Cobb, L.K.; He, F.J. Impact of Color-Coded and Warning Nutrition Labelling Scheme: A Systematic Review and Network Meta-Analysis. PLoS Med. 2021, 18, e1003765, doi:10.1371/journal.pmed.1003765.

137. Guadalupe Hernandez-Nava, L.; Egnell, M.; Alberto Aguilar-Salinas, C.; Angel Cordova-Villalobos, J.; Armando Barriguete-Melendez, J.; Pettigrew, S.; Hercberg, S.; Julia, C.; Galan, P. Impact of different front-of-package nutrition label on foods according to their nutritional quality: a comparative study in Mexico. SALUD PUBLICA Mex. 2019, 61, 609–618, doi:10.21149/10318.

138. Jauregui, A.; White, C.M.; Vanderlee, L.; Hall, M.; Contreras-Manzano, A.; Nieto, C.; Sacks, G.; Thrasher, J.F.; Hammond, D.; Barquera, S. Impact of Front-of-Package Label on the Perceived Healthfulnes of a Sweetened Fruit Drink: A Randomised Experiment in Five Countries. PUBLIC Health Nutr. 2022, 25, 1094–1104, doi:10.1017/S1368980021004535.

139. Scarpelli Dourado, D.Q.; Gomes Ramires, T.; Araneda, J.; Pinheiro Fernandes, A.C. Impact of front-of-package labeling on foods purchase pattern in Chile. Nutr. Hosp. 2021, 38, 358–365, doi:10.20960/nh.03311.

140. Arrua, A.; Curutchet, M.R.; Rey, N.; Barreto, P.; Golovchenko, N.; Sellanes, A.; Velazco, G.; Winokur, M.; Gimenez, A.; Ares, G. Impact of Front-of-Package Nutrition Information and Label Design on Children's Choices of Two Snack Foods: Comparison of Warnings and the Traffic-Light. APPETITE 2017, 116, 139–146, doi:10.1016/j.appet.2017.04.012.

141. Jauregui, A.; Vargas-Meza, J.; Nieto, C.; Contreras-Manzano, A.; Alejandro-Torres, N.Z.; Tolentino-Mayo, L.; Hall, M.; Barquera, S. Impact of Front-of-Package Nutrition Label on Consumer Purchasing Intention: A Randomized Experiment in Low- and Middle-Income Mexican Adults. BMC PUBLIC Health 2020, 20, 463, doi:10.1186/s12889-020-08549-0.

142. Taillie, L.S.; Bercholz, M.; Prestemon, C.E.; Higgins, I.C.A.; Grummon, A.H.; Hall, M.; Jaacks, L.M. Impact of Taxes and Warning Label on Red Meat Purchases among Us Consumer: A Randomized Controlled Trial. PLoS Med. 2023, 20, e1004284, doi:10.1371/journal.pmed.1004284.

143. Fernandes, T.F. da C.; Ferreira, N.B.; Campagnoli, R.R.; Gomes, F.; Braga, F.; David, I.A.; Lobo, I. Impact of Textual Warnings on Emotional Brain Responses to Ultra-Processed Foods Product. Front. Nutr. 2022, 9, 895317, doi:10.3389/fnut.2022.895317.

144. Franco-Arellano, B.; Vanderlee, L.; Ahmed, M.; Oh, A.; L'Abbe, M. Influence of Front-of-Package Labelling and Regulated Nutrition Claims on Consumer' Perception of Product Healthfulnes and Purchase Intention: A Randomized Controlled Trial. APPETITE 2020, 149, 104629, doi:10.1016/j.appet.2020.104629.

145. Kim, M.K.; Kwak, H.S. Influence of Functional Information on Consumer Liking and Consumer Perception Related to Health Claims for Blueberry Functional Beverages. Int. J. Foods Sci. Technol. 2015, 50, 70–76, doi:10.1111/ijfs.12627.

146. Senna Prates, S.M.; Reis, I.A.; Urquizar Rojas, C.F.; Spinillo, C.G.; Anastacio, L.R. Influence of Nutrition Claims on Different Model of Front-of-Package Nutritional Labeling in Supposedly Health Foods: Impact on the Understanding of Nutritional Information, Healthfulnes Perception, and Purchase Intention of Brazilian Consumer. Front. Nutr. 2022, 9, 921065, doi:10.3389/fnut.2022.921065.

147. Tortora, G.; Machin, L.; Ares, G. Influence of Nutritional Warnings and Other Label Features on Consumer' Choices: Results from an Eye-Tracking Study. Foods Res. Int. 2019, 119, 605–611, doi:10.1016/j.foodsres.2018.10.038.

148. Temple, J.L.; Ziegler, A.M.; Epstein, L.H. Influence of Price and Labeling on Energy Drink Purchasing in an Experimental Convenience Store. J. Nutr. Educ. Behav. 2016, 48, 54-+, doi:10.1016/j.jneb.2015.08.007.

149. Tortora, G.; Ares, G. Influence of Time Orientation on Foods Choices: Case Study with Cookie Label. Foods Res. Int. 2018, 106, 706–711, doi:10.1016/j.foodsres.2018.01.045.

150. Lima, M.; de Alcantara, M.; Ares, G.; Deliza, R. It Is Not All about Information! Sensory Experience Overrides the Impact of Nutrition Information on Consumer' Choices of Sugar-Reduced Drink. Foods Qual. Prefer. 2019, 74, 1–9, doi:10.1016/j.foodsqual.2018.12.013.

151. Magalhaes Silva, E.B.; Martins Vieira, V.R.; Rodrigues de Oliveira Goncalves, S.A.; Takahashi, J.A.; Bello de Araujo, R.L. Labeling analysis of mixed fruit and vegetable drink called detox. Vigil. Sanit. EM DEBATE-Soc. Cienc. Tecnol. 2021, 9, 130–136, doi:10.22239/2317-269x.01458.

152. da Silva, S.A.; de Moura Dias, M.R.; Pinto de Castro Ferreira, T.A. Labeling foods product for breastfeeding infant and toddlers. Rev. Nutr.-Braz. J. Nutr. 2008, 21, 185–194, doi:10.1590/S1415-52732008000200007.

153. Saavedra-Garcia, L.; Taboada-Ramirez, X.; Hernandez-Vasquez, A.; Diez-Canseco, F. Marketing Techniques, Health, and Nutritional Claims on Processed Foods and Beverages before and after the Implementation of Mandatory Front-of-Package Warning Label in Peru. Front. Nutr. 2022, 9, 1004106, doi:10.3389/fnut.2022.1004106.

154. Bullon-Vela, V.; Sayon-Orea, C.; Gomez-Donoso, C.; Martinez, J.; Martinez-Gonzalez, M.A.; Bes-Rastrollo, M. Mortality Prediction of the Nutrient Profile of the Chilean Front-of-Package Warning Label: Results from the Seguimiento Universidad de Navarra Prospective Cohort Study. Front. Nutr. 2022, 9, 951738, doi:10.3389/fnut.2022.951738.

155. Miller, C.; Dono, J.; Wright, K.; Pettigrew, S.; Wakefield, M.; Coveney, J.; Wittert, G.; Roder, D.; Durkin, S.; Martin, J.; et al. "No Child or Adult Would Ever Probably Choose to Have 16 Teaspoons of Sugar": A Preliminary Study of Parents' Responses to Sugary Drink Warning Label Options. NUTRIENTS 2022, 14, 4173, doi:10.3390/nu14194173.

156. Delgado Zegarra, J.; da Silva Gomes, F. Nutrient Profile of Foods Product Exempted from the Application of Front-of- Package Warnings during the First Stage of the Health Eating Law in Peru: Case Study. Rev. Panam. SALUD PUBLICA-PAN Am. J. PUBLIC Health 2021, 45, e153, doi:10.26633/RPSP.2021.153.

157. Khosravi, A.; Bassetti, E.; Yuen-Esco, K.; Sy, N.Y.; Kane, R.; Sweet, L.; Zehner, E.; Pries, A.M. Nutrient Profiles of Commercially Produced Complementary Foods Available in Burkina Faso, Cameroon, Ghana, Nigeria and Senegal. NUTRIENTS 2023, 15, 2279, doi:10.3390/nu15102279.

158. Khandpur, N.; Swinburn, B.; Monteiro, C.A. Nutrient-Based Warning Label May Help in the Pursuit of Health Diet. OBESITY 2018, 26, 1670–1671, doi:10.1002/oby.22318.

159. Gerke, S. “Nutrition Facts Label” for Artificial Intelligence/Machine Learning-Based Medical Devices-The Urgent Need for Labeling Standards. GEORGE Wash. LAW Rev. 2023, 90, 78–85.

160. Alonso-Dos-Santos, M.; Quilodrin Ulloa, R.; Salgado Quintana, A.; Vigueras Quijada, D.; Farias Nazel, P. Nutrition Labeling Scheme and the Time and Effort of Consumer Processing. SusTAINABILITY 2019, 11, 79, doi:10.3390/su11041079.

161. Mora-Plazas, M.; Gomez, L.F.; Miles, D.R.; Parra, D.C.; Taillie, L.S. Nutrition Quality of Packaged Foods in Bogota, Colombia: A Comparison of Two Nutrient Profile Model. NUTRIENTS 2019, 11, 1011, doi:10.3390/nu11051011.

162. Cabrera, M.; Machin, L.; Arrua, A.; Antunez, L.; Curutchet, M.R.; Gimenez, A.; Ares, G. Nutrition Warnings as Front-of-Package Label: Influence of Design Features on Healthfulnes Perception and Attentional Capture. PUBLIC Health Nutr. 2017, 20, 3360–3371, doi:10.1017/S136898001700249X.

163. Annunziata, A.; Pomarici, E.; Vecchio, R.; Mariani, A. Nutritional Information and Health Warnings on Wine Label: Exploring Consumer Interest and Preference. APPETITE 2016, 106, 58–69, doi:10.1016/j.appet.2016.02.152.

164. Scott, K. Nutritional Labeling, Communication Design, and Relevance. Front. Commun. 2023, 8, 1125575, doi:10.3389/fcomm.2023.1125575.

165. Meza-Hernandez, M.; Villarreal-Zegarra, D.; Saavedra-Garcia, L. Nutritional Quality of Foods and Beverages Offered in Supermarkets of Lima According to the Peruvian Law of Health Eating. NUTRIENTS 2020, 12, 1508, doi:10.3390/nu12051508.

166. Ares, G.; Aschemann-Witzel, J.; Curutchet, M.R.; Antunez, L.; Machin, L.; Vidal, L.; Martinez, J.; Gimenez, A. Nutritional Warnings and Product Substitution or Abandonment: Policies Implications Derived from a Repeated Purchase Simulation. Foods Qual. Prefer. 2018, 65, 40–48, doi:10.1016/j.foodsqual.2017.12.001.

167. Cominato, L.; Di Biagio, G.F.; Lellis, D.; Franco, R.R.; Mancini, M.C.; de Melo, M.E. Obesity Prevention: Strategies and Challenges in Latin America. Curr. Obes. Rep. 2018, 7, 97–104, doi:10.1007/s13679-018-0311-1.

168. Contreras-Manzano, A.; Jauregui, A.; Vargas-Meza, J.; Nieto, C.; Granich-Armenta, A.; Aleman Escobar, M. de L.; G-Olvera, A.; Cruz-Casarrubias, C.; Munguia, A.; Barquera, S. Objective Understanding of Front of Pack Warning Label

among Mexican Children of Public Elementary Schools. A Randomized Experiment. Nutr. J. 2022, 21, 47, doi:10.1186/s12937-022-00791-z.

169. Egnell, M.; Talati, Z.; Hercberg, S.; Pettigrew, S.; Julia, C. Objective Understanding of Front-of-Package Nutrition Label: An International Comparative Experimental Study across 12 Countries. NUTRIENTS 2018, 10, 1542, doi:10.3390/nu10101542.

170. Egnell, M.; Talati, Z.; Galan, P.; Andreeva, V.A.; Vandevijvere, S.; Gombaud, M.; Dreano-Trecant, L.; Hercberg, S.; Pettigrew, S.; Julia, C. Objective Understanding of the Nutri-Score Front-of-Package Label by European Consumer and Its Effect on Foods Choices: An Online Experimental Study. Int. J. Behav. Nutr. Phys. Act. 2020, 17, 146, doi:10.1186/s12966-020-01053-z.

171. Kontopoulou, L.; Karpetas, G.; Fradelos, E.C.; Papathanasiou, I.V.; Malli, F.; Papagiannis, D.; Mantzaris, D.; Fialon, M.; Julia, C.; Gourgoulianis, K.I. Online Consumer Survey Comparing Different Front-of-Package Label in Greece. NUTRIENTS 2022, 14, 46, doi:10.3390/nu14010046.

172. Falbe, J.; Musicus, A.A.; Sigala, D.M.; Roberto, C.A.; Solar, S.E.; Lemmon, B.; Sorscher, S.; Nara, D.; Hall, M. Online RCT of Icon Added-Sugar Warning Label for Restaurant Menus. Am. J. Prev. Med. 2023, 65, 101–111, doi:10.1016/j.amepre.2023.02.007.

173. Pomeranz, J.L. Outstanding Questions In First Amendment Law Related To Foods Labeling Disclosure Requirements For Health. Health Aff. (Millwood) 2015, 34, 1986–1992, doi:10.1377/hlthaff.2015.0616.

174. Sambra, V.; Lopez-Arana, S.; Caceres, P.; Abrigo, K.; Collinao, J.; Espinoza, A.; Valenzuela, S.; Carvajal, B.; Prado, G.; Peralta, R.; et al. Overuse of Non-Caloric Sweeteners in Foods and Beverages in Chile: A Threat to Consumer' Free Choices? Front. Nutr. 2020, 7, 68, doi:10.3389/fnut.2020.00068.

175. Bopape, M.; Taillie, L.S.; Swart, R. Perceived Effect of Warning Label on Parental Foods Purchasing and Drivers of Foods Selection among South African Parents-An Exploratory Study. Front. PUBLIC Health 2022, 10, 939937, doi:10.3389/fpubh.2022.939937.

176. Campos-Nonato, I.; Cervantes-Armenta, M.A.; Pacheco-Miranda, S.; Quezada-Sanchez, A.D.; Contreras-Manzano, A.; Barquera, S.; Vargas-Meza, J. Perception and Understanding of Guideline Daily Amount and Warning Labeling among Mexican Adults during the Law Modification Period. NUTRIENTS 2022, 14, 3403, doi:10.3390/nu14163403.

177. Casas-Caruajulca, E.; Muguruz-Sanchez, L.J.; Calizaya-Milla, Y.E.; Saintila, J. Perception of frontal foods labeling, purchase and consumption of ultra-

processed foods during the COVID-19 quarantine: A cross-sectional study in the Peruvian population. Rev. ESPANOLA Nutr. HUMANA Diet. 2021, 25, e1473, doi:10.14306/renhyd.25.S2.1473.

178. Bandeira, L.M.; Pedroso, J.; Toral, N.; Gubert, M.B. Performance and Perception on Front-of-Package Nutritional Labeling Model in Brazil. Rev. SAUDE PUBLICA 2021, 55, 19, doi:10.11606/s1518-8787.2021055002395.

179. Ang, F.J.L.; Agrawal, S.; Finkelstein, E.A. Pilot Randomized Controlled Trial Testing the Influence of Front-of-Package Sugar Warning Label on Foods Demand. BMC PUBLIC Health 2019, 19, 164, doi:10.1186/s12889-019-6496-8.

180. Andreeva, V.A.; Egnell, M.; Stos, K.; Przygoda, B.; Talati, Z.; Touvier, M.; Galan, P.; Hercberg, S.; Pettigrew, S.; Julia, C. Polish Consumer' Understanding of Different Front-of-Package Foods Label: A Randomized Experiment. foods 2022, 11, 134, doi:10.3390/foods11010134.

181. Basto-Abreu, A.; Torres-Alvarez, R.; Reyes-Sanchez, F.; Gonzalez-Morales, R.; Canto-Osorio, F.; Arantxa Colchero, M.; Barquera, S.; Rivera, J.A.; Barrientos-Gutierrez, T. Predicting Obesity Reduction after Implementing Warning Label in Mexico: A Modeling Study. PLoS Med. 2020, 17, e1003221, doi:10.1371/journal.pmed.1003221.

182. Bassetti, E.; Khosravi, A.; Pries, A.M. Prevalence of Front-of-Package Warning Signs among Commercial Complementary Foods in Seven High and Upper Middle-Income Countries. NUTRIENTS 2023, 15, 1629, doi:10.3390/nu15071629.

183. Aliaga-Ortega, L.; Adasme-Berrios, C.; Mendez, C.; Soto, C.; Schnettler, B. Processed Foods Choices Based on the Theory of Planned Behavior in the Context of Nutritional Warning Label. Br. Foods J. 2019, 121, 3266–3280, doi:10.1108/BFJ-10-2018-0695.

184. Cooper, J.M. Product Reformulation - Can Sugar Be Replaced in Foods? Int. SUGAR J. 2012, 114, 642–645.

185. Ares, G.; Aschemann-Witzel, J.; Curutchet, M.R.; Antunez, L.; Machin, L.; Vidal, L.; Gimenez, A. Product Reformulation in the Context of Nutritional Warning Label: Exploration of Consumer Preference towards Foods Concepts in Three Foods Categories. Foods Res. Int. 2018, 107, 669–674, doi:10.1016/j.foodsres.2018.03.021.

186. Bera, O.P.; Saleem, S.M.; Bhattacharya, S. Promoting Healthier Diet in India through "Front of Package Foods Labeling." INDIAN J. COMMUNITY Health 2021, 33, 25–29, doi:10.47203/IJCH.2021.v33i01.004.

187. Ares, G.; Antunez, L.; Curutchet, M.R.; Galicia, L.; Natero, V.; Gimenez, A.; Otterbring, T. Qualitative Exploration of the Reasons for Not Using Nutritional Warnings after Policies Implementation in Uruguay. Health Promot. Int. 2023, 38, daac174, doi:10.1093/heapro/daac174.

188. Younes, M.; Aquilina, G.; Castle, L.; Degen, G.; Engel, K.-H.; Fowler, P.J.; Fernandez, M.J.F.; Fuerst, P.; Gundert-Remy, U.; Guertler, R.; et al. Re-Evaluation of Erythritol (E 968) as a Foods Additive. EFSA J. 2023, 21, e8430, doi:10.2903/j.efsa.2023.8430.

189. Younes, M.; FAF, E.F.S.A.; Aquilina, G.; Castle, L.; Degen, G.; Engel, K.-H.; Fowler, P.J.; Fernandez, M.J.F.; Furst, P.; Gundert-Remy, U.; et al. Re-Evaluation of Erythritol (E 968) as a Foods Additive. EFSA J. 2023, 21, e8430, doi:10.2903/j.efsa.2023.8430.

190. Campos-Nonato, I.; Vargas-Meza, J.; Nieto, C.; Carolina Ariza, A.; Barquera, S. Reducing Sodium Consumption in Mexico: A Strategy to Decrease the Morbidity and Mortality of Cardiovascular Disease. Front. PUBLIC Health 2022, 10, 857818, doi:10.3389/fpubh.2022.857818.

191. Devia, G.; Forli, S.; Vidal, L.; Curutchet, M.R.; Ares, G. References to Home-Made and Natural Foods on the Label of Ultra-Processed Foods Increase Healthfulnes Perception and Purchase Intention: Insights for Policies Making. Foods Qual. Prefer. 2021, 88, 104110, doi:10.1016/j.foodsqual.2020.104110.

192. Lowery, C.M.; Mora-Plazas, M.; Gomez, L.F.; Popkin, B.; Taillie, L.S. Reformulation of Packaged Foods and Beverages in the Colombian Foods Supply. NUTRIENTS 2020, 12, 3260, doi:10.3390/nu12113260.

193. Saavedra-Garcia, L.; Meza-Hernandez, M.; Diez-Canseco, F.; Taillie, L.S. Reformulation of Top-Selling Processed and Ultra-Processed Foods and Beverages in the Peruvian Foods Supply after Front-of-Package Warning Label Policies. Int. J. Environ. Res. Public. Health 2023, 20, 424, doi:10.3390/ijerph20010424.

194. Centurion, M.; Machin, L.; Ares, G. Relative Impact of Nutritional Warnings and Other Label Features on Cereal Bar Healthfulnes Evaluations. J. Nutr. Educ. Behav. 2019, 51, 850–856, doi:10.1016/j.jneb.2019.01.021.

195. Fernando Gomez, L.; Fernanda Parra, M.; Ivan Lucumi, D.; Camila Bermudez, L.; Jose Calderon, A.; Mantilla, J.; Mora-Plazas, M. Relevance of Political Actions Aimed at Guaranteeing a Health and Sufficient Diet During and After the COVID-19 Pandemic in the Context of Latin America. Univ. MEDICA 2021, 62, doi:10.11144/Javeriana.umed62-3.rapd.

196. Correa, T.; Fierro, C.; Reyes, M.; Carpentier, F.R.D.; Taillie, L.S.; Corvalan, C. Responses to the Chilean Law of Foods Labeling and Advertising: Exploring

Knowledge, Perception and Behavior of Mothers of Young Children. Int. J. Behav. Nutr. Phys. Act. 2019, 16, 21, doi:10.1186/s12966-019-0781-x.

197. Spink, J.; Singh, J.; Singh, S.P. Review of Package Warning Label and Their Effect on Consumer Behaviour with Insights to Future Anticounterfeit Strategy of Label and Communication System. Packag. Technol. Sci. 2011, 24, 469–484, doi:10.1002/pts.947.

198. Duran, A.C.; Ricardo, C.Z.; Mais, L.A.; Bortoletto Martins, A.P. Role of Different Nutrient Profiling Model in Identifying Targeted Foods for Front-of-Package Foods Labelling in Brazil. PUBLIC Health Nutr. 2021, 24, 1514–1525, doi:10.1017/S1368980019005056.

199. Packer, J.; Russell, S.J.; Ridout, D.; Conolly, A.; Jessop, C.; Viner, R.M.; Croker, H. Secondary Outcomes of a Front-of-Package-Labelling Randomised Controlled Experiment in a Representative British Sample: Understanding, Ranking Speed and Perception. NUTRIENTS 2022, 14, 2188, doi:10.3390/nu14112188.

200. Madilo, F.K.; Owusu-Kwarteng, J.; Kunadu, A.P.-H.; Tano-Debrah, K. Self-Reported Use and Understanding of Foods Label Information among Tertiary Education Students in Ghana. Foods CONTROL 2020, 108, 106841, doi:10.1016/j.foodscont.2019.106841.

201. Ares, G.; Antunez, L.; Otterbring, T.; Curutchet, M.R.; Galicia, L.; Moratorio, X.; Bove, I. Sick, Salient and Full of Salt, Sugar and Fat: Understanding the Impact of Nutritional Warnings on Consumer' Associations through the Salience Bias. Foods Qual. Prefer. 2020, 86, 103991, doi:10.1016/j.foodsqual.2020.103991.

202. Mehlhose, C.; Risius, A. Signs of Warning: Do Health Warning Messages on Sweets Affect the Neural Prefrontal Cortex Activity? NUTRIENTS 2020, 12, 3903, doi:10.3390/nu12123903.

203. Lee, B.Y.; Ferguson, M.C.; Hertenstein, D.L.; Adam, A.; Zenkov, E.; Wang, P.I.; Wong, M.S.; Gittelsohn, J.; Mui, Y.; Brown, S.T. Simulating the Impact of Sugar Sugar Sweetened Beverages Warning Label in Three Cities. Am. J. Prev. Med. 2018, 54, 197–204, doi:10.1016/j.amepre.2017.11.003.

204. Sisti, J.S.; Prasad, D.; Niederman, S.; Mezzacca, T.A.; Anekwe, A.V.; Clapp, J.; Farley, S.M. Sodium-Intake of Menu Items in New York City Chain Restaurants Following Enforcement of the Sodium Warning Icon Rule, 2015-2017. PLoS ONE 2023, 18, e0274648, doi:10.1371/journal.pone.0274648.

205. Bopape, M.; Taillie, L.S.; Frank, T.; Murukutla, N.; Cotter, T.; Majija, L.; Swart, R.; Scott, J.A.; Scott, J.A.; Scott, J.A. South African Consumer' Perception of Front-of-Package Warning Label on Unhealth Foods and Drink. PLoS ONE 2021, 16, e0257626, doi:10.1371/journal.pone.0257626.

206. Duran Aguero, S.; Silva Ocampo, P.; Gimenez Sanchez, J.; Fleta Sanchez, Y.; Moya Tilleria, J. Stages of Change in the Purchase of Packaged Foods after Phase 1 of the Implementation of the New Foods Policies in Chile 2017. NUTRITION 2020, 71, 110593, doi:10.1016/j.nut.2019.110593.

207. Duran Aguero, S.; Silva Rojas, J.; Caichac, A.; Araneda, J.; Willson Rojas, W.; Buhring, R.; Pacheco, V.; Encina, C.; Ahumada, D.; Fernandez-Salamanca, M.; et al. Stages of Change in the Purchase of Ultra-Processed Snacks among University Students after the Implementation of the Chilean Foods Law; a Multi-Center Study. Arch. Latinoam. Nutr. 2020, 70, 263–268, doi:10.37527/2020.70.4.004.

208. Corvalan, C.; Reyes, M.; Garmendia, M.L.; Uauy, R. Structural Responses to the Obesity and Non-Communicable Disease Epidemic: The Chilean Law of Foods Labeling and Advertising. Obes. Rev. 2013, 14, 79–87, doi:10.1111/obr.12099.

209. Corvalan, C.; Reyes, M.; Garmendia, M.L.; Uauy, R. Structural Responses to the Obesity and Non-Communicable Disease Epidemic: Update on the Chilean Law of Foods Labelling and Advertising. Obes. Rev. 2019, 20, 367–374, doi:10.1111/obr.12802.

210. Yen, C.; Huang, Y.-L.; Chung, M.; Chen, Y.-C. Sugar Content and Warning Criteria Evaluation for Popular Sugar Sugar Sweetened Beverages in Taipei, Taiwan. NUTRIENTS 2022, 14, 3339, doi:10.3390/nu14163339.

211. Liu, C.-H.; Wong, T.-C.; Chung, M.; Bai, C.-H.; Chen, Y.-C. Sugar Labeling Information and Online Marketing Strategies for Hand-Shaken Tea Drink in Northern Taiwan. Front. Nutr. 2023, 10, 1273713, doi:10.3389/fnut.2023.1273713.

212. Grummon, A.H.; Taillie, L.S.; Golden, S.D.; Hall, M.; Ranney, L.M.; Brewer, N.T. Sugar Sugar Sweetened Beverages Health Warnings and Purchases: A Randomized Controlled Trial. Am. J. Prev. Med. 2019, 57, 601–610, doi:10.1016/j.amepre.2019.06.019.

213. Lowery, C.M.; Saavedra-Garcia, L.; Diez-Canseco, F.; Cardenas, M.K.; Miranda, J.J.; Taillie, L.S. Sugar Sugar Sweetened Beverages Purchases in Urban Peru before the Implementation of Taxation and Warning Label Policies: A Baseline Study. BMC PUBLIC Health 2022, 22, 2389, doi:10.1186/s12889-022-14762-w.

214. Pomeranz, J.L.; Mozaffarian, D.; Micha, R. Sugar Sugar Sweetened Beverages Warning Policies in the Broader Legal Context: Health and Safety Warning Laws and the First Amendment. Am. J. Prev. Med. 2020, 58, 783–788, doi:10.1016/j.amepre.2020.01.006.

215. Grummon, A.H.; Hall, M. Sugary Drink Warnings: A Meta-Analysis of Experimental Studies. PLoS Med. 2020, 17, e1003120, doi:10.1371/journal.pmed.1003120.

216. Campbell, N.; Legowski, B.; Legetic, B.; Ferrante, D.; Nilson, E.; Campbell, C.; L'Abbe, M. Targets and Timelines for Reducing Salt in Processed Foods in the Americas. J. Clin. Hypertens. 2014, 16, 619–623, doi:10.1111/jch.12379.

217. Acton, R.B.; Jones, A.C.; Kirkpatrick, S.I.; Roberto, C.A.; Hammond, D. Taxes and Front-of-Package Label Improve the Healthiness of Beverage and Snack Purchases: A Randomized Experimental Marketplace. Int. J. Behav. Nutr. Phys. Act. 2019, 16, 46, doi:10.1186/s12966-019-0799-0.

218. Taillie, L.S.; Chauvenet, C.; Grummon, A.H.; Hall, M.; Waterlander, W.; Prestemon, C.E.; Jaacks, L.M. Testing Front-of-Package Warnings to Discourage Red Meat Consumption: A Randomized Experiment with Us Meat Consumer. Int. J. Behav. Nutr. Phys. Act. 2021, 18, 114, doi:10.1186/s12966-021-01178-9.

219. Baidal, J.W.A.; Nichols, K.; Charles, N.; Chernick, L.; Duong, N.; Finkel, M.A.; Falbe, J.; Valeri, L. Text Messages to Curb Sugar Sugar Sweetened Beverages Consumption among Pregnant Women and Mothers: A Mobile Health Randomized Controlled Trial. NUTRIENTS 2021, 13, 4367, doi:10.3390/nu13124367.

220. Pettigrew, S.; Jongenelis, M.I.; Talati, Z.; Dana, L.M.; Hercberg, S.; Julia, C. The Ability of Five Different Front-of-Package Label to Assist Australian Consumer to Identify Health versus Unhealth Foods. Aust. N. Z. J. PUBLIC Health 2023, 47, doi:10.1016/j.anzjph.2022.100017.

221. Velasco Vizcaino, F.; Velasco, A. The Battle between Brands and Nutritional Label: How Brand Familiarity Decreases Consumer' Alertness toward Traffic Light Nutritional Label. J. Bus. Res. 2019, 101, 637–650, doi:10.1016/j.jbusres.2019.02.054.

222. Sandoval, L.A.; Carpio, C.E.; Sanchez-Plata, M. The Effect of "Traffic-Light" Nutritional Labelling in Carbonated Soft Drink Purchases in Ecuador. PLoS ONE 2019, 14, e0222866, doi:10.1371/journal.pone.0222866.

223. Donnelly, G.E.; Zatz, L.Y.; Svirsky, D.; John, L.K. The Effect of Graphic Warnings on Sugary-Drink Purchasing. Psychol. Sci. 2018, 29, 1321–1333, doi:10.1177/0956797618766361.

224. Billich, N.; Blake, M.R.; Backholer, K.; Cobcroft, M.; Li, V.; Peeters, A. The Effect of Sugar Sugar Sweetened Beverages Front-of-Package Label on Drink Selection, Health Knowledge and Awarenes: An Online Randomised Controlled Trial. APPETITE 2018, 128, 233–241, doi:10.1016/j.appet.2018.05.149.

225. Vanderlee, L.; Franco-Arellano, B.; Ahmed, M.; Oh, A.; Lou, W.; L'Abbe, M. The Efficacy of "high in" Warning Label, Health Star and Traffic Light Front-of-Package Labelling: An Online Randomised Control Trial. PUBLIC Health Nutr. 2021, 24, 62–74, doi:10.1017/S1368980020003213.

226. Kanter, R.; Reyes, M.; Swinburn, B.; Vandevijvere, S.; Corvalan, C. The Foods Supply Prior to the Implementation of the Chilean Law of Foods Labeling and Advertising. NUTRIENTS 2019, 11, 52, doi:10.3390/nu11010052.

227. Mantzari, E.; Pechey, R.; Codling, S.; Sexton, O.; Hollands, G.J.; Marteau, T.M. The Impact of "on-Pack" Pictorial Health Warning Label and Calorie Information Label on Drink Choices: A Laboratory Experiment. APPETITE 2020, 145, 104484, doi:10.1016/j.appet.2019.104484.

228. Hall, M.; Lazard, A.J.; Grummon, A.H.; Mendel, J.R.; Taillie, L.S. The Impact of Front-of-Package Claims, Fruit Images, and Health Warnings on Consumer' Perception of Sugar-Sweetened Fruit Drink: Three Randomized Experiments. Prev. Med. 2020, 132, 105998, doi:10.1016/j.ypmed.2020.105998.

229. Hammond, D.; Acton, R.B.; Goodman, S. The Impact of Health Warnings for Sugar Sugar Sweetened Beverages on Consumer Perception of Advertising. PUBLIC Health Nutr. 2021, 24, 4737–4749, doi:10.1017/S1368980021001257.

230. Hall, M.; Grummon, A.H.; Higgins, I.C.A.; Lazard, A.J.; Prestemon, C.E.; Avendano-Galdamez, M.I.; Taillie, L.S. The Impact of Pictorial Health Warnings on Purchases of Sugary Drink for Children: A Randomized Controlled Trial. PLoS Med. 2022, 19, e1003885, doi:10.1371/journal.pmed.1003885.

231. Acton, R.B.; Hammond, D. The Impact of Price and Nutrition Labelling on Sugary Drink Purchases: Results from an Experimental Marketplace Study. APPETITE 2018, 121, 129–137, doi:10.1016/j.appet.2017.11.089.

232. Hamlin, R.; McNeill, L. The Impact of the Australasian "Health Star Rating", Front-of-Package Nutritional Label, on Consumer Choices: A Longitudinal Study. NUTRIENTS 2018, 10, 906, doi:10.3390/nu10070906.

233. Colchero, M.A.; Paraje, G.; Popkin, B. The Impacts on Foods Purchases and Tax Revenues of a Tax Based on Chile's Nutrient Profiling Model. PLoS ONE 2021, 16, e0260693, doi:10.1371/journal.pone.0260693.

234. Roberto, C.A.; Wong, D.; Musicus, A.A.; Hammond, D. The Influence of Sugar Sugar Sweetened Beverages Health Warning Label on Parents' Choices. PEDIATRICS 2016, 137, e20153185, doi:10.1542/peds.2015-3185.

235. VanEpps, E.M.; Roberto, C.A. The Influence of Sugar Sugar Sweetened Beverages Warnings A Randomized Trial of Adolescents' Choices and Beliefs. Am. J. Prev. Med. 2016, 51, 664–672, doi:10.1016/j.amepre.2016.07.010.

236. Pandav, C.; Taillie, L.S.; Miles, D.R.; Hollingsworth, B.A.; Popkin, B. The WHO South-East Asia Region Nutrient Profile Model Is Quite Appropriate for India: An

Exploration of 31,516 Foods Product. NUTRIENTS 2021, 13, 2799, doi:10.3390/nu13082799.

237. Richter, A.P.C.; Duffy, E.W.; Higgins, I.C.A.; Barrington, C.; Martin, S.L.; Aquilina, K.H.; Avendano-Galdamez, M.I.; Hall, M. Toddler Milk Perception and Responses to Front-of-Package Claims and Product Warnings: A Qualitative Study of Caregivers of Toddlers. J. Acad. Nutr. Diet. 2023, 123, 1568-+, doi:10.1016/j.jand.2023.06.281.

238. Machin, L.; Aschemann-Witzel, J.; Rosa Curutchet, M.; Gimenez, A.; Ares, G. Traffic Light System Can Increase Healthfulnes Perception: Implications for Policies Making. J. Nutr. Educ. Behav. 2018, 50, 668–674, doi:10.1016/j.jneb.2018.03.005.

239. Cui, J.; Yan, R.; Astell-Burt, T.; Gong, E.; Zheng, L.; Li, X.; Zhang, J.; Xiang, L.; Ye, L.; Hu, Y.; et al. Types and Aspects of Front-of-Package Labeling Preferred by Parents: Insights for Policies Making in China. NUTRIENTS 2022, 14, 800, doi:10.3390/nu14040800.

240. Nieto, C.; Jauregui, A.; Contreras-Manzano, A.; Arillo-Santillan, E.; Barquera, S.; White, C.M.; Hammond, D.; Thrasher, J.F. Understanding and Use of Foods Labeling System among Whites and Latinos in the United States and among Mexicans: Results from the International Foods Policies Study, 2017. Int. J. Behav. Nutr. Phys. Act. 2019, 16, 87, doi:10.1186/s12966-019-0842-1.

241. Galan, P.; Egnell, M.; Salas-Salvado, J.; Babio, N.; Pettigrew, S.; Hercberg, S.; Julia, C. Understanding of different front-of-package label by the Spanish population: Results of a comparative study. Endocrinol. DIABETES Nutr. 2020, 67, 122–129, doi:10.1016/j.endinu.2019.03.013.

242. Sagaceta-Mejia, J.; Tolentino-Mayo, L.; Cruz-Casarrubias, C.; Nieto, C.; Barquera, S. Understanding of Front of Package Nutrition Label: Guideline Daily Amount and Warning Label in Mexicans with Non-Communicable Disease. PLoS ONE 2022, 17, e0269892, doi:10.1371/journal.pone.0269892.

243. Ares, G.; Machin, L.; Vidal, L.; Otterbring, T.; Aschemann-Witzel, J.; Curutchet, M.R.; Bove, I. Uruguayan Citizens' Perception of Messages to Promote Health Eating Through the Use of Nutritional Warnings. J. Nutr. Educ. Behav. 2020, 52, 918–927, doi:10.1016/j.jneb.2020.05.012.

244. Machin, L.; Alcaire, F.; Antunez, L.; Gimenez, A.; Curutchet, M.R.; Ares, G. Use of Nutritional Warning Label at the Point of Purchase: An Exploratory Study Using Self-Reported Measures and Eye-Tracking. APPETITE 2023, 188, 106634, doi:10.1016/j.appet.2023.106634.

245. Hall, M.; Higgins, I.C.A.; Grummon, A.H.; Lazard, A.J.; Prestemon, C.E.; Sheldon, J.M.; Taillie, L.S. Using a Naturalistic Store Laboratory for Clinical Trials of Point-of-Sale Nutrition Policies and Intervention: A Feasibility and Validation Study. Int. J. Environ. Res. Public. Health 2021, 18, 8764, doi:10.3390/ijerph18168764.

246. Uribe, R.; Manzur, E.; Cornejo, C. Varying the Number of FOP Warnings on Hedonic and Utilitarian Foods Product: Evidence from Chile. J. Foods Prod. Mark. 2020, 26, 123–143, doi:10.1080/10454446.2020.1738971.

247. Ares, G.; Antunez, L.; Curutchet, M.R.; Gimenez, A. Warning Label as a Policies Tool to Encourage Healthier Eating Habits. Curr. Opin. Foods Sci. 2023, 51, 101011, doi:10.1016/j.cofs.2023.101011.

248. Boncinelli, F.; Gerini, F.; Pagnotta, G.; Alfnes, F. Warning Label on Junk Foods: Experimental Evidence. Int. J. Consum. Stud. 2017, 41, 46–53, doi:10.1111/ijcs.12312.

249. Popova, L.; Nonnemaker, J.; Taylor, N.; Bradfield, B.; Kim, A. Warning Label on Sugar Sugar Sweetened Beverages: An Eye Tracking Approach. Am. J. Health Behav. 2019, 43, 406–419, doi:10.5993/AJHB.43.2.16.

250. Leung, C.W.; Wolfson, J.A.; Hsu, R.; Soster, K.; Mangan, S.; Falbe, J. Warning Label Reduce Sugar Sugar Sweetened Beverages Intake among College Students. J. Nutr. 2021, 151, 179–185, doi:10.1093/jn/nxaa305.

251. Arrua, A.; Machin, L.; Curutchet, M.R.; Martinez, J.; Antunez, L.; Alcaire, F.; Gimenez, A.; Ares, G. Warnings as a Directive Front-of-Package Nutrition Labelling Scheme: Comparison with the Guideline Daily Amount and Traffic-Lights. PUBLIC Health Nutr. 2017, 20, 2308–2317, doi:10.1017/S1368980017000866.

252. Adasme-Berrios, C.; Aliaga-Ortega, L.; Schnettler, B.; Sanchez, M.; Pinochet, C.; Lobos, G. What Dimensions of Risk Perception Are Associated with Avoidance of Buying Processed Foods with Warning Label? NUTRIENTS 2020, 12, 2987, doi:10.3390/nu12102987.

253. Correa, T.; Fierro, C.; Reyes, M.; Taillie, L.S.; Carpentier, F.R.D.; Corvalan, C. Why Don't You [Government] Help Us Make Healthier Foods More Affordable Instead of Bombarding Us with Label? Maternal Knowledge, Perception, and Practices after Full Implementation of the Chilean Foods Labelling Law. Int. J. Environ. Res. Public. Health 2022, 19, 4547, doi:10.3390/ijerph19084547.

254. Miller, C.; Wright, K.; Dono, J.; Pettigrew, S.; Wakefield, M.; Coveney, J.; Wittert, G.; Roder, D.; Durkin, S.; Martin, J.; et al. "You Can't Just Eat 16 Teaspoons of Sugar so Why Would You Drink 16 Teaspoons' Worth of Sugar?": A Qualitative Study of Young Adults' Reactions to Sugary Drink Warning Label. BMC PUBLIC Health 2022, 22, 1241, doi:10.1186/s12889-022-13648-1.

255. Gratale, S.K.; Jeong, M.; Sidhu, A.; Safi, Z.; Strasser, A.A.; Delnevo, C.D.; Wackowski, O.A. Young Adults’ Cigarillo Risk Perception, Attention to Warning Label and Perception of Proposed Pictorial Warnings: A Focus Group Study. BMJ OPEN 2022, 12, e061064, doi:10.1136/bmjopen-2022-061064.
